# PRECISE-seq reveals disease-relevant TCR repertoires with phenotypic plasticity

**DOI:** 10.1084/jem.20251779

**Published:** 2026-05-12

**Authors:** Shibo Liu, Guanghao Liang, Yayun Yang, Yanyang Shi, Lihui Dong, Yue Zhao, Boyuan Mei, Jun Wang, Feng Lin, Yilin Li, Wenxin Dong, Chengyang Liu, Yuhui Cao, Dali Han, Peng R. Chen, Meng Michelle Xu

**Affiliations:** 1 New Cornerstone Science Laboratory, Synthetic and Functional Biomolecules Center, Key Laboratory of Bioorganic Chemistry and Molecular Engineering of Ministry of Education, Beijing National Laboratory for Molecular Sciences, College of Chemistry and Molecular Engineering, Peking University, Beijing, China; 2 State Key Laboratory of Molecular Oncology, School of Basic Medical Sciences, Institute for Immunology, Tsinghua University, Beijing, China; 3 Department of Computational Biology, Key Laboratory of RNA Science and Engineering, China National Center for Bioinformation, Beijing Institute of Genomics, Chinese Academy of Sciences, Beijing, China; 4 College of Future Technology, Sino-Danish College, University of Chinese Academy of Sciences, Beijing, China; 5 RootPath, Inc., Guangzhou, China; 6 Department of Pathology, New York University Grossman School of Medicine, New York, NY, USA; 7 The Laura and Isaac Perlmutter Cancer Center, New York University Langone Health, New York, NY, USA; 8 Institute for Stem Cell and Regeneration, Chinese Academy of Sciences, Beijing, China; 9 Peking-Tsinghua Center for Life Sciences, Peking University, Beijing, China; 10 Shenzhen Bay Laboratory, Shenzhen, China

## Abstract

Linking T cell phenotypes with antigen specificity and functional avidity is critical for understanding in vivo immune responses in infection and cancer. Here, we develop PRECISE-seq, a method that integrates multi-omics T cell analysis with contact-dependent **pr**oximity lab**e**ling for rapid s**c**reening of d**is**ease-relevant T c**e**ll repertoires, and for linking the relative TCR avidity with T cell phenotypes at single-cell resolution. PRECISE-seq accurately retrieves CMV-specific clonotypes from human peripheral blood and quantitatively measures functional avidity in physiological contexts. We find that high-potency CMV-specific T cells preferentially acquire an exhausted phenotype. In tumors, polyclonal tumor-reactive CD8^+^ T cells predominantly differentiate into a protumor Ly49^+^ regulatory state (T_Ly49_), characterized by inhibitory killer cell lectin-like receptor expression and originating from effector memory T cells along a trajectory distinct from exhaustion. Notably, PD-1 blockade reduces T_Ly49_ formation and promotes effector revival, which correlates with responsiveness to immunotherapy. Together, PRECISE-seq enables high-resolution mapping of TCR potency and T cell phenotype, revealing a regulatory axis shaping T cell fate in tumors.

## Teaser

Liu et al. develop PRECISE-seq, a single-cell platform linking TCR specificity and avidity to T cell phenotypes in vivo. They reveal that high-potency antiviral T cells become exhausted, while tumor-reactive CD8^+^ T cells acquire a regulatory Ly49^+^ state that is restrained by PD-1 blockade, resulting in effector revival within tumors.

## Graphical Abstract

**Figure:**
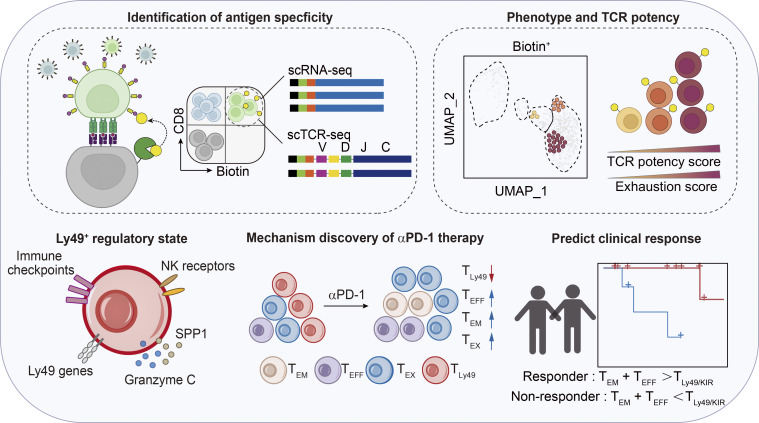

## Introduction

T cells are central to immune surveillance against infections and cancer (Cox et al., 2019; Waldman et al., 2020). T cell–based immunotherapies have achieved remarkable clinical success, relying on specific interactions between T cell receptors (TCRs) and peptide–major histocompatibility complexes (pMHCs). Alongside TCR specificity, another important consideration for clinical development is TCR potency (Chan et al., 2023; Klebanoff et al., 2023; Zhao et al., 2022), which refers to the ability of TCRs to initiate T cell responses to naturally processed peptides displayed on target cells. TCR potency is typically measured through functional avidity assays that preserve physiological parameters, including TCR-pMHC density, affinity, and coreceptor engagement. Importantly, TCR potency shapes T cell fate and functional phenotypes in vivo (Huang et al., 2010; Liu et al., 2014). Linking TCR specificity and potency with phenotypic states is therefore essential for understanding T cell responses during immunotherapies and optimizing TCR-based therapies (Oliveira and Wu, 2023).

Current approaches for identifying disease-associated TCRs rely on either pMHC multimer staining (Ma et al., 2021; Newell et al., 2013) or in vitro functional screening of TCR clonotypes (Arnaud et al., 2022; Chandran et al., 2022; Dijkstra et al., 2018; Lowery et al., 2022; Rosenberg and Restifo, 2015). While pMHC multimers allow simultaneous assessment of antigen specificity and phenotype, their reliance on predefined antigens limits multiplex capacity, and binding affinity alone often poorly predicts functional outcomes (Sibener et al., 2018). Functional assays based on coculture of T cells and antigen-presenting cells (APCs) circumvent predefined epitope requirements but require prolonged in vitro culture, leading to loss of native phenotypic information (Dijkstra et al., 2018; Lowery et al., 2022). Thus, there remains a need for high-throughput platforms capable of simultaneously decoding antigen specificity, quantifying functional avidity under physiological conditions, and preserving endogenous T cell phenotypes. Contact-dependent proximity labeling enables mapping of live T cell–target interactions within confined spatial contexts, offering a potential solution (Liu et al., 2020; Pasqual et al., 2018). However, current strategies suffer from variable labeling efficiency due to heterogeneous substrate expression on prey cells (Liu et al., 2020; Nakandakari-Higa et al., 2024; Pasqual et al., 2018), and nonspecific background beyond the immunological synapse (Oakley et al., 2022; Zhou and Zou, 2021), limiting sensitivity for detecting differences in TCR potency.

Here, we present PRECISE-seq, a contact-dependent proximity labeling sequencing platform that integrates Sortase A (SrtA)–catalyzed ligation to simultaneously profile antigen specificity, TCR potency, and phenotypic landscape. In PRECISE-seq, T cells are uniformly modified with acceptor peptides (APs) via chemical conjugation to ensure consistent substrate availability. Spatially restricted SrtA-catalyzed ligation confines signal deposition to the immunological synapse, minimizing off-target background. PRECISE-seq robustly identifies antigen-specific T cells, with labeling intensity quantitatively reflecting TCR potency. Application to human cytomegalovirus (CMV)-specific T cells revealed that high-potency clonotypes preferentially exhibit an exhaustion phenotype. In tumors, PRECISE-seq identified a Ly49^+^ CD8^+^ T cell (T_Ly49_) state characterized by inhibitory killer cell lectin-like receptor expression and immunoregulatory activity in suppressing host antitumor response. PD-1 blockade rejuvenates tumor-specific T cells, driving a transition from the T_Ly49_ state to an effector-like state that is associated with improved immunotherapy responsiveness in colorectal cancer (CRC), hepatocellular carcinoma (HCC), and melanoma. Thus, PRECISE-seq enables rapid characterization of in vivo T cell responsiveness across diseases, facilitating the advancement of T cell–based therapies against infections and cancers.

## Results

### Design and development of PRECISE-seq to decipher the intercellular interactions

**Figure 1.**
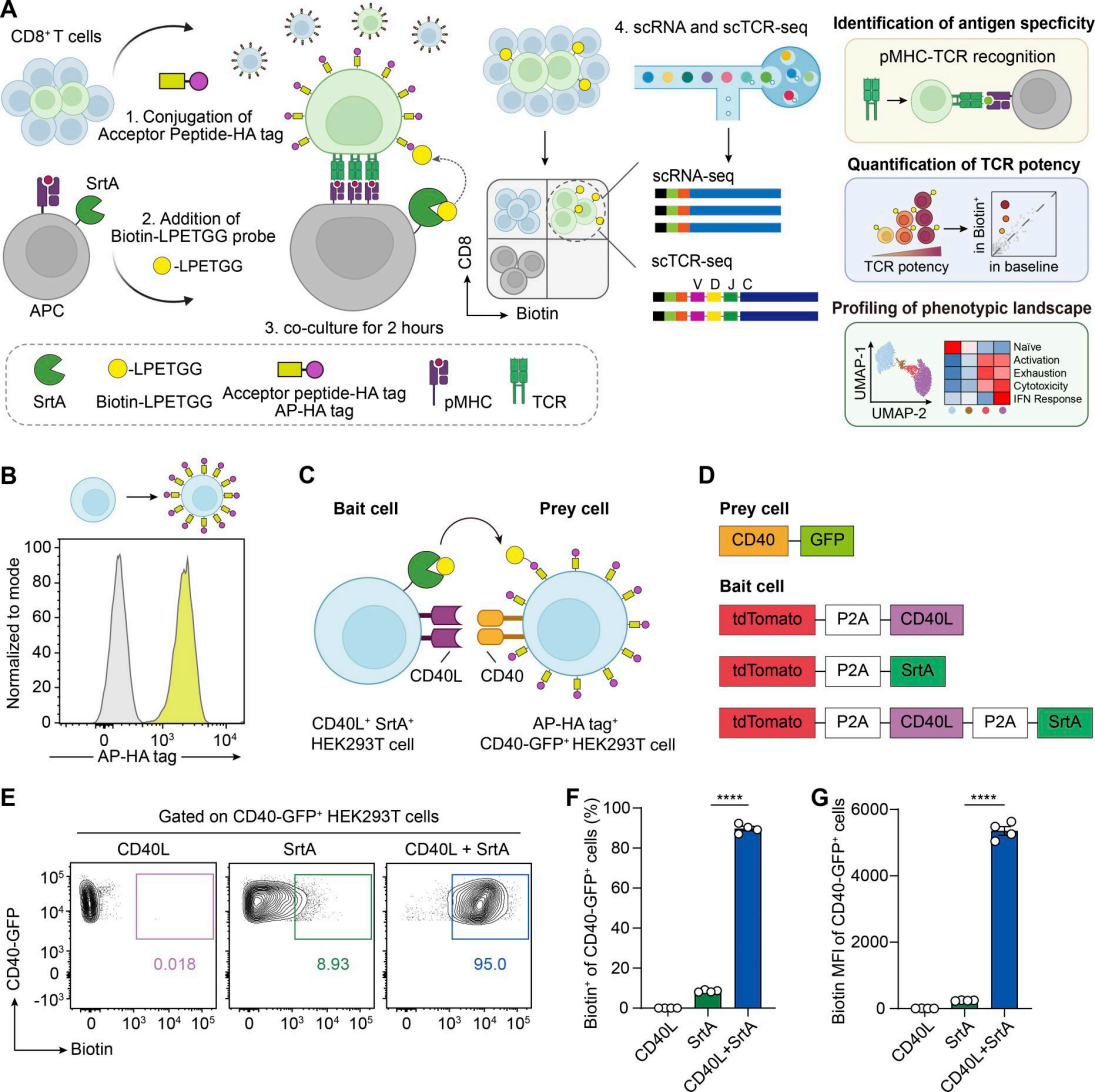
**Design and validation of PRECISE-seq. (A)** Schematic illustration of PRECISE-seq. SrtA was genetically overexpressed on the surface of APCs, and AP-HA tag was conjugated on T cells. Cognate antigen-specific T cells were covalently labeled with Biotin probes via SrtA-mediated proximity labeling and subsequently isolated for paired scRNA-seq and scTCR-seq. PRECISE-seq characterizes antigen specificity, TCR potency, and phenotypic landscape for comprehensive T cell analysis. **(B)** Histograms showing the abundance of the AP-HA tag on HEK293T cells following two consecutive chemical reactions. **(C)** Schematic representation of PRECISE-seq for capturing CD40-CD40L recognition-dependent intercellular interactions between HEK293T cells. **(D)** Plasmid constructs used in E–G. The plasmid construct transfected into prey cells encodes CD40 protein and a C-terminal fused GFP reporter. The plasmid construct transfected into bait cells encodes tdTomato, CD40L, and SrtA protein, separated by a bicistronic gene. The SrtA protein is anchored on the cell membrane through the pDisplay system. Bait cells expressing either SrtA or CD40L alone are utilized as the negative control. **(E)** Flow cytometry analysis of Biotin signals on AP-HA tag^+^ CD40-GFP fusion protein^+^ HEK293T cells after coculturing with bait cells expressing CD40L, SrtA, or both proteins. **(F)** Bar plot showing the frequency of Biotin^+^ cells in AP-HA tag^+^ CD40-GFP^+^ HEK293T cells after coculturing with the indicated bait cells. **(G)** Bar plot showing the MFI of Biotin on AP-HA tag^+^ CD40-GFP^+^ HEK293T cells after coculturing with the indicated bait cells. Error bars represent the mean ± SEM. Each dot represents a different technical replicate; *n* = 4; statistical significance is determined by unpaired, two-tailed Student’s *t* test; **** represents P < 0.0001. MFI, median fluorescence intensity.

**Figure S1.**
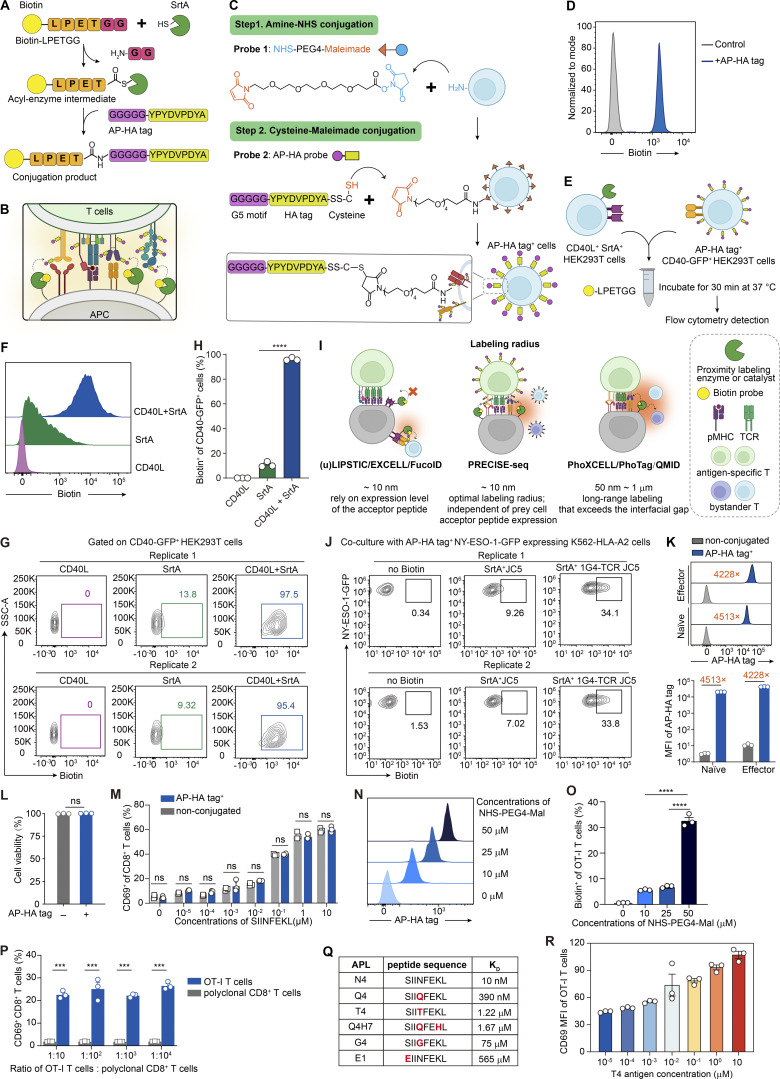
**Development of PRECISE-seq to precisely decipher intercellular interactions. (A)** Schematic representation of sortagging. SrtA specifically recognizes the Biotin-LPETGG probe and forms an acyl-enzyme intermediate. A nucleophilic attack by the oligo-glycine at the N terminus resolves the intermediate, resulting in the covalent ligation of Biotin-LPETGG to the AP-HA tag. **(B)** Schematic illustration showing the covalent transfer of the Biotin-LPETGG probe to the proximal AP-HA tag conjugated to the opposite T cells across the immune synapse by SrtA. **(C)** Strategy for the attachment of the AP-HA tag to the T cell surface by a two-step chemical reaction. First, the prey cell surface is modified with maleimide groups (orange) through the conjugation of a primary amine with NHS-PEG4-maleimide. The AP-HA tag (peptide sequence: GGGGGYPYDVPDYASSC) is then attached to the maleimide-modified prey cells through cysteine–maleimide conjugation. The HA tag is appended to the AP tag to facilitate the detection of labeling efficiency in this two-step chemical reaction process. **(D)** Representative histograms showing the Biotin signals of HEK293T cells conjugated with the AP-HA tag and incubated with purified SrtA protein in the presence of the Biotin-LPETGG probe at 37°C for 30 min. **(E)** Experimental setup for assessing the capacity of PRECISE-seq in capturing CD40 and CD40L recognitions within HEK293T cells. Bait cells (SrtA^+^CD40L^+^ HEK293T) and prey cells (CD40-GFP^+^ HEK293T) tagged with AP-HA were mixed in the presence of Biotin-LPETGG probes for 30 min, followed by flow cytometry analysis. **(F)** Representative histograms showing the Biotin signals of the AP-HA tag^+^ CD40-GFP^+^ HEK293T cells after coculturing with bait cells expressing CD40L, SrtA, or both proteins. **(G)** Flow cytometry analysis of Biotin signals on AP-HA tag^+^ CD40-GFP^+^ HEK293T cells after coculture with bait cells expressing CD40L, SrtA, or both proteins. Data from two independent replicates are shown. **(H)** Bar plot showing the frequency of Biotin^+^ cells in AP-HA tag^+^ CD40-GFP^+^ HEK293T cells after coculture with bait cells expressing CD40L, SrtA, or both proteins in three independent replicates. **(I)** Comparison of labeling radius across different contact-dependent proximity labeling strategies. **(J)** Representative flow cytometry plot depicting Biotin signal expression on AP-HA tag^+^ NY-ESO-1-GFP^+^ K562-HLA-A2 cells, which endogenously present NY-ESO-1 peptides, after coculture with SrtA^+^ JC5 cells expressing either a cognate antigen-specific 1G4-TCR or no TCR. **(K)** Histograms and bar plots showing the abundance of the chemically conjugated AP-HA tag on naïve OT-I T cells versus effector OT-I T cells. The abundance of the AP-HA tag was measured by flow cytometry via HA tag staining. The fold change quantifies the relative surface expression levels of HA-tagged molecules between experimental and control groups, calculated as the ratio of MFI in AP-HA–tagged cells to nonconjugated controls. All values were derived from triplicate experimental measurements. **(L)** Bar plot illustrating the viability of OT-I T cells subsequent to chemical conjugation with the AP-HA tag. The cell viability was measured by flow cytometry via 7-AAD staining. **(M)** Bar plot depicting CD69 expression on AP-HA tag^+^ or nonconjugated OT-I T cells stimulated by MC38 tumor cells, which has been pulsed with serially diluted concentrations of the OVA_257–264_ peptide. **(N)** Histograms showing the abundance of the AP-HA tag on OT-I T cells following incubation with titrated concentrations of NHS-PEG4-Mal ranging from 0 to 50 μM. **(O)** Bar plots depicting the frequency of Biotin^+^ OT-I T cells labeled by B16-OVA-SrtA cells, with OT-I T cells expressing distinct levels of the AP-HA tag as indicated in N. **(P)** Bar plots showing the frequency of CD69^+^ cells within CD45.1^+^ OT-I T cells and CD45.2^+^ polyclonal CD8^+^ T cells, respectively. CD45.1^+^ OT-I T cells were mixed with CD45.2^+^ polyclonal CD8^+^ T cells at different ratios, ranging from 1:10 to 1:10,000. Following conjugation with the AP-HA tag, the T cell mixture was cocultured with B16-OVA-SrtA tumor cells for 2 h. **(Q)** Table summarizing the peptide sequence and potency of APLs. **(R)** Bar plots showing the CD69 MFI of OT-I T cells after incubation with MC38-SrtA cells preloaded with different doses of T4 peptide (SIITFEKL). Error bars represent the mean ± SEM. Each dot represents a different technical replicate; *n* = 3; statistical significance is determined by unpaired, two-tailed Student’s *t* test; ns represents P > 0.05; *** represents P < 0.001; **** represents P < 0.0001. MFI, median fluorescence intensity.

PRECISE-seq comprises two primary processes: the proximity-based labeling of T cells during antigen recognition, followed by high-throughput single-cell sequencing of the labeled T cells. For the labeling step, PRECISE-seq employs SrtA-mediated transpeptidation (sortagging), wherein engineered SrtA on the cell membrane of bait cells covalently transfers the Biotin-LPETGG probe to proximal APs on neighboring prey cells (Fig. 1 A and Fig. S1, A and B). The sensitivity and unbiased nature of PRECISE-seq are highly dependent on the level of the AP present on the surfaces of neighboring prey cells. To establish a precise and unbiased contact-dependent labeling method, we developed a two-step chemical reaction to introduce a homogeneous level of the AP (G5 tag) on the surfaces of prey cells. The prey cells were modified with the maleimide group through conjugation of primary amine with NHS-PEG4-maleimide, followed by cysteine–maleimide conjugation to attach the AP-HA tag (peptide sequence: GGGGGYPYDVPDYASSC) (Fig. S1 C). We then assessed the conjugation efficiency of the two-step chemical reaction. Flow cytometry data demonstrated that the two-step chemical reaction successfully yielded uniform and abundant AP-HA tag display on the cell surface, enabling efficient recognition and labeling by SrtA (Fig. 1 B and Fig. S1 D).

To assess the capability of PRECISE-seq in deciphering physical intercellular interactions, we established a cellular contact system based on CD40 and CD40L recognition within HEK293T cells (Fig. 1, C and D). We co-expressed SrtA and CD40L on the surface of HEK293T cells, which served as bait cells (SrtA^+^CD40L^+^ HEK293T), and chemically attached prey cells (CD40-GFP^+^ HEK293T) with the AP-HA tag. The two populations were mixed in the presence of the biotinylated SrtA substrate (Biotin-LPETGG) for 30 min, followed by flow cytometry analysis to detect Biotin signals (Fig. S1 E). We observed efficient labeling of prey cells (CD40-GFP^+^ HEK293T) by PRECISE-seq when cocultured with SrtA^+^CD40L^+^ HEK293T cells, as evidenced by a robust Biotin signal (Fig. 1, E–G; and Fig. S1, F–H). In contrast, minimal Biotin signals were detected on prey cells cocultured with SrtA^+^ HEK293T cells lacking CD40L expression. Overall, these results indicate that PRECISE-seq effectively records intercellular interactions mediated by specific ligand–receptor pairs.

### PRECISE-seq enables the accurate identification of T cell antigen specificity

**Figure 2.**
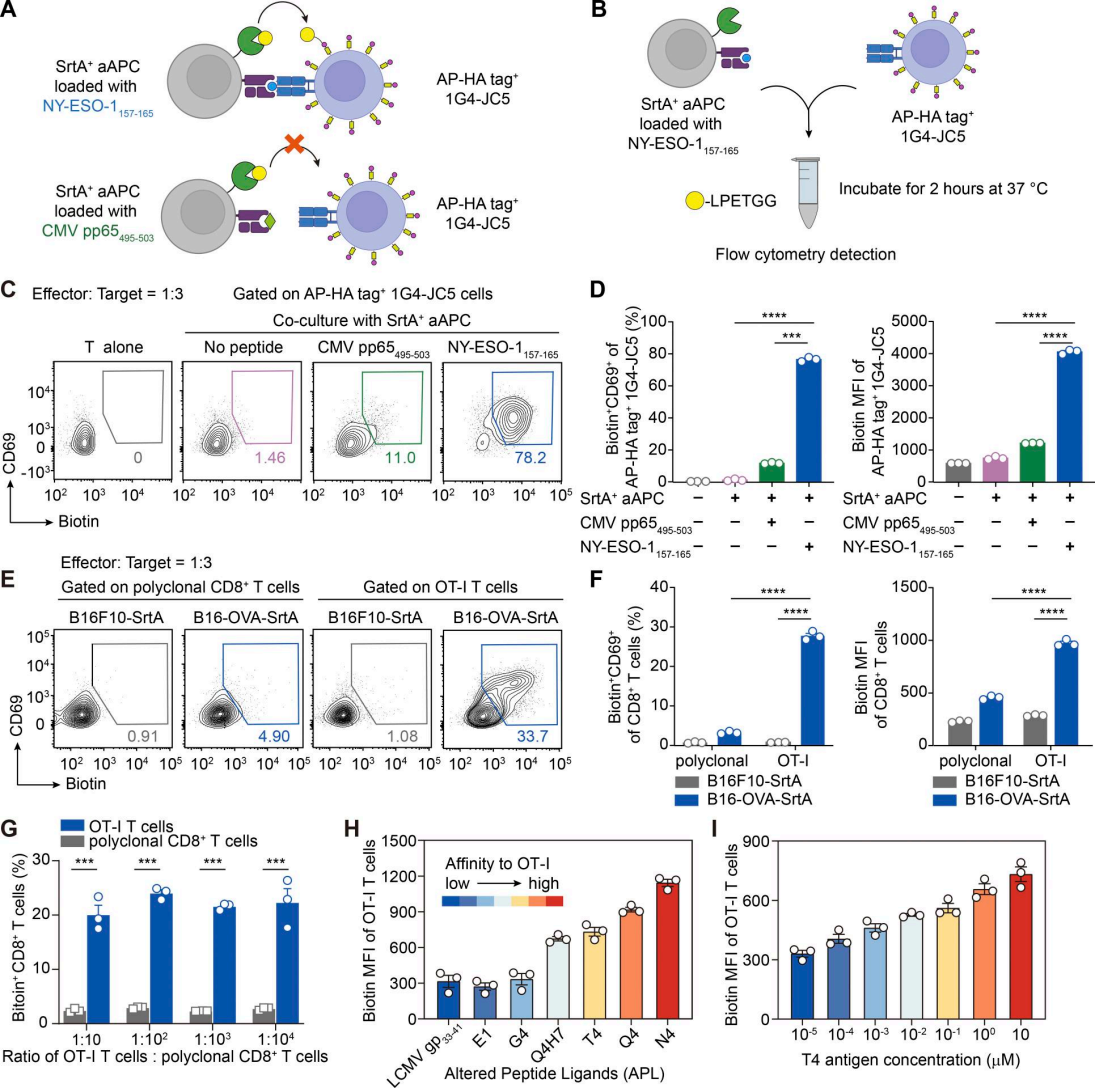
**PRECISE-seq accurately interrogates T cell antigen specificity and TCR potency. (A)** Schematic illustration of PRECISE-seq for assessing the antigen specificity of T cells. 1G4-TCR can recognize the NY-ESO-1_157–165_ presented by SrtA^+^ aAPC (SrtA^+^HLA-A2^+^ K562 cells) but not the irrelevant CMV pp65_495–503_. The AP-HA tag^+^ 1G4-JC5 cells were specifically activated and labeled by SrtA^+^ aAPCs presenting NY-ESO-1_157–165_ instead of CMV pp65_495–503_. **(B)** Experimental setup for assessing the antigen specificity of T cells via PRECISE-seq. AP-HA tag^+^ 1G4-JC5 cells were incubated with SrtA^+^ aAPCs presenting cognate NY-ESO-1_157–165_ in the presence of Biotin-LPETGG probe for 2 h at 37°C and then subjected to flow cytometry analysis. The frequencies of Biotin^+^ AP-HA tag^+^ 1G4-JC5 cells cocultured with irrelevant CMV pp65_495–503_-pulsed or mock-pulsed SrtA^+^ aAPC were utilized as the negative control. **(C)** Representative flow plot showing the expression of the activation marker CD69 and Biotin signals on AP-HA tag^+^ 1G4-JC5 cells after coculture with SrtA^+^ aAPCs presenting either cognate or the irrelevant peptide. **(D)** Bar plots showing the frequency of CD69^+^Biotin^+^ cells (left) and Biotin MFI (right) of AP-HA tag^+^ 1G4-JC5 cells from C. **(E)** Representative flow plot showing the CD69 expression and Biotin signals of CD45.1^+^ OT-I T cells upon incubation with B16-OVA-SrtA or B16F10-SrtA tumor cells. Polyclonal CD8^+^ T cells derived from CD45.2 C57BL/6 mice were utilized as the negative control. **(F)** Bar plots showing the frequency of CD69^+^Biotin^+^ cells (left) and the Biotin MFI (right) of OT-I T cells and antigen-irrelevant polyclonal CD8^+^ T cells. **(G)** Bar plots showing the frequency of Biotin^+^ cells within CD45.1^+^ OT-I T cells and CD45.2^+^ polyclonal CD8^+^ T cells. The CD45.1^+^ OT-I T cells and CD45.2^+^ polyclonal CD8^+^ T cells decorated with the AP-HA tag were mixed at different ratios, ranging from 1:10 to 1:10,000, and subsequently cocultured with B16-OVA-SrtA tumor cells for 2 h. **(H)** Bar plots showing the Biotin MFI of OT-I T cells following 2 h of incubation with MC38-SrtA tumor cells preloaded with 10 μM APLs derived from the original N4 peptide (SIINFEKL). **(I)** Bar plots showing the Biotin MFI of OT-I T cells after incubation with MC38-SrtA tumor cells preloaded with different doses of the T4 peptide (SIITFEKL). Error bars represent the mean ± SEM. Each dot represents a different technical replicate; *n* = 3; statistical significance is determined by unpaired, two-tailed Student’s *t* test; *** represents P < 0.001; **** represents P < 0.0001. MFI, median fluorescence intensity.

Given the relatively short labeling radius of PRECISE-seq (Lin et al., 2023, *Preprint*; Oakley et al., 2022; Zhang et al., 2023; Zhou and Zou, 2021), we inferred that this method can accurately record the specific recognition between a live T cell and its target cell (Fig. S1 I). For this purpose, we employed the specific recognition between 1G4-TCR and NY-ESO-1_157–165_ presented by HLA-A2 (Jäger et al., 1998; Li et al., 2005) (Fig. 2 A). We cloned 1G4-TCR into a TCR-deficient Jurkat T cell line (1G4-JC5 cells). Additionally, we constructed an artificial APC (aAPC), which was established on the K562 cell line with stable expression of HLA-A2 and SrtA. Subsequently, we cocultured AP-HA tag^+^ 1G4-JC5 cells with SrtA^+^ aAPCs presenting NY-ESO-1_157–165_, in the presence of Biotin-LPETGG substrate for 2 h (Fig. 2 B). We observed functional activation of 1G4-JC5 cells, evidenced by the upregulation of the TCR downstream signaling marker CD69. Notably, nearly all CD69^+^ 1G4-JC5 cells were labeled with Biotin (Fig. 2, C and D). Conversely, when incubated with either irrelevant CMV pp65_495–503_-pulsed or mock-pulsed SrtA^+^ aAPC, few 1G4-JC5 cells displayed Biotin signals. In addition, PRECISE efficiently labeled K562-HLA-A2 cells that endogenously present NY-ESO-1 peptides when cocultured with SrtA^+^ 1G4-JC5 cells, but not with SrtA^+^ JC5 cells. This confirms the method’s specificity and efficacy in labeling APCs that present endogenous peptides (Fig. S1 J). These findings underscore the capacity of PRECISE-seq to accurately decode antigen specificity, with the Biotin signals correlating with functional activation.

We then extended the application of PRECISE-seq to determine the antigen specificity of primary CD8^+^ T cells. We evaluated the efficiency of AP-HA tag conjugation using naïve T cells (T_N_) and effector T cells (T_EFF_), two representative T cell subsets with distinct transcriptional profiles. Flow cytometry data indicated that the abundance of the AP-HA tag on T_EFF_ was comparable to that on T_N_ (Fig. S1 K), suggesting that PRECISE-seq mitigates potential labeling discrepancies arising from diverse transcriptional activities across cellular states. Additionally, the cellular viability and antigen sensitivity of T cells remained unaffected after the conjugation of the AP-HA tag (Fig. S1, L and M).

Subsequently, we cocultured SrtA-expressing B16-OVA tumor cells with primary OT-I T cells conjugated with the AP-HA tag, whose TCRs specifically recognize the OVA antigen. Flow cytometry analysis revealed that the majority of CD69^+^ OT-I T cells activated by cognate OVA antigen were simultaneously labeled with Biotin probes by PRECISE-seq (Fig. 2, E and F). Conversely, minimal Biotin signals were detected on OT-I T cells when cocultured with B16F10-SrtA cells, confirming the ability of PRECISE-seq to capture primary antigen-specific T cells. Of note, the abundance of AP-HA tags on T cells was critical for labeling efficiency, as sub-optimal conjugation significantly reduced downstream labeling (Fig. S1, N and O). To assess the sensitivity of PRECISE-seq, we mixed target OT-I T cells with antigen-irrelevant polyclonal CD8^+^ T cells from wild-type mice in various ratios, ranging from 1:10 to 1:10,000 (OT-I: polyclonal CD8^+^ T cells). Analysis of Biotin signals on OT-I T cells demonstrated consistent performance, even when the proportion of antigen-specific T cells was as low as 0.01% (Fig. 2 G and Fig. S1 P), indicating the sensitivity of PRECISE-seq in detecting low-frequency antigen-specific T cells.

### Quantifying TCR potency by PRECISE-seq to evaluate the T cell responsiveness

The ex vivo assessment of TCR potency within the cellular context provides superior metrics for evaluating T cell responsiveness. However, its application is limited by relatively low throughput and a tedious screening and validation process. To assess the capacity of PRECISE-seq in quantifying TCR potency, we utilized an established model employing altered peptide ligands (APLs) to modulate the strength of APC–T cell interactions across a range of antigenic potencies (Daniels et al., 2006). The original ligand OVA_257–264_ (SIINFEKL, N4) is most potent in stimulating OT-I cells, while the remaining five APLs (Q4, T4, Q4H7, G4, and E1), derived from N4, were listed in a descending order based on their potency in stimulating OT-I cells (Fig. S1 Q). A gradual decrease in labeling intensity of OT-I T cells corresponding to the recognition of APLs with potencies ranging from high to low was observed (Fig. 2 H). We further examined whether PRECISE-seq could resolve dose-dependent differences by exposing T cells to increasing doses of APLs. The Biotin signals detected by PRECISE-seq faithfully reflected the binding strength of TCR-pMHC interactions and demonstrated consistency with T cell activation, as measured by CD69 upregulation (Fig. 2 I and Fig. S1 R). These data indicate that PRECISE-seq can accurately quantify TCR potency guided by the strength of TCR-pMHC interactions.

### PRECISE-seq accurately retrieves CMV-specific T cell clonotypes from peripheral blood

**Figure 3.**
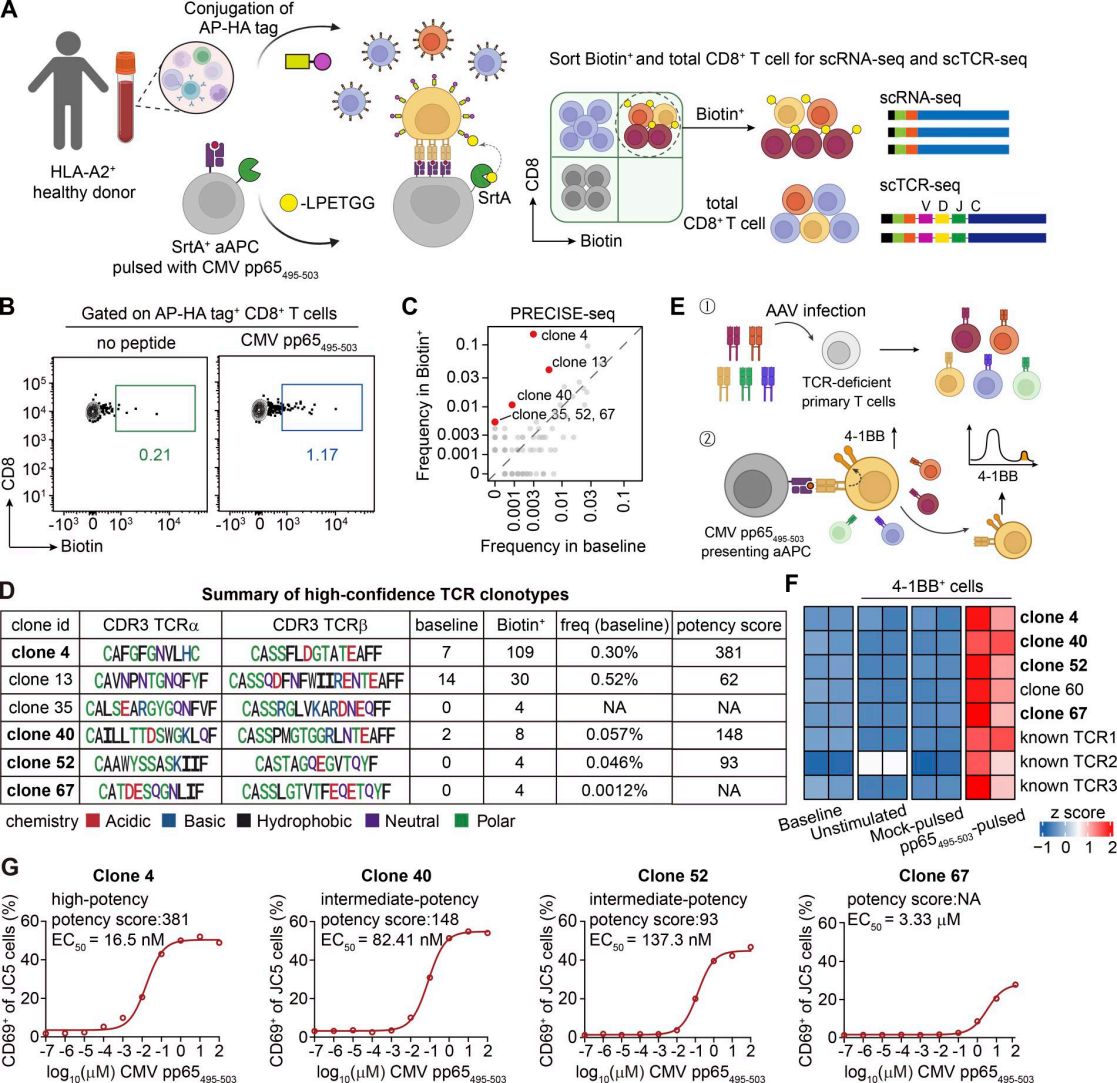
**PRECISE-seq enables rapid identification of CMV-specific T cells from human peripheral blood. (A)** Schematic illustration of PRECISE-seq for simultaneous interrogation of antigen specificity, TCR potency, and transcriptional profiles of CMV-specific T cells from PBMCs. PBMCs are derived from an HLA-A2^+^ CMV-seropositive donor. The AP-HA tag^+^ PBMCs were incubated with SrtA^+^ aAPCs presenting the CMV pp65_495–503_ epitope in the presence of Biotin-LPETGG probe. After 2 h of incubation, both Biotin^+^ and total CD8^+^ T cells without AP-HA tag conjugation were isolated and further subjected to paired scRNA-seq and scTCR-seq. **(B)** Flow cytometry analysis of Biotin signals on AP-HA tag^+^ CD8^+^ T cells from PBMCs in the assay for identification of T cells specific to the CMV pp65_495–503_ epitope. AP-HA tag^+^ CD8^+^ T cells cocultured with mock-pulsed SrtA^+^ aAPC were served as the negative control to determine the Biotin^+^ population for PRECISE-seq. **(C)** Frequencies of TCR clonotypes in the baseline (x axis) group and the Biotin^+^ group (y axis) in PRECISE-seq. High-confidence CMV-specific clonotypes (red) were defined by Fisher’s exact test (FDR < 0.05 and OR > 4). **(D)** Summary table of high-confidence CMV-specific TCR clonotypes identified by PRECISE-seq. The frequency of each clonotype was determined by the detected TCRβ frequency in bulk TCR-seq. TCR clonotypes validated by the TCR-pMHC activation screening assay were marked in bold. The potency score for each clonotype was calculated based on the fold change in frequency between the Biotin^+^ group and the baseline group (determined by bulk TCR-seq) and was then adjusted according to the distribution of irrelevant TCRs (Materials and methods). The abbreviation “NA” signifies “not available.” **(E)** Schematic illustration showing the antigen specificity validation of candidate T cell clonotypes identified by PRECISE-seq. The candidate TCRs were synthesized and transfected into TCR-deficient primary T cells. The antigen specificity of TCR was then assessed by 4-1BB upregulation under cognate CMV pp65_495–503_ stimulation. **(F)** Heatmap showing the z scores of frequencies for the CMV-specific TCR clonotypes identified via the TCR-pMHC activation screening assay. Engineered T cells were stimulated with either the CMV pp65_495–503_-pulsed or mock-pulsed APCs. Following stimulation, 4-1BB^+^ cells were isolated for TCR repertoire analysis. CMV-specific clonotypes were defined as those significantly enriched (FDR < 0.05 and OR > 4) in the CMV pp65_495–503_-pulsed group compared with both the mock-pulsed group and the baseline group. **(G)** Experimental validation of the TCR potency score by in vitro CMV pp65_495–503_ peptide stimulation. TCR-deficient Jurkat T cells (JC5 cells) were transduced with CMV-specific TCR and stimulated with serially diluted CMV pp65_495–503_ peptide presented by K562-HLA-A2 cells. The expression of CD69 on JC5 cells was detected by flow cytometry, and the EC_50_ of each TCR was calculated based on the proportion of CD69^+^ JC5 cells.

**Figure S2.**
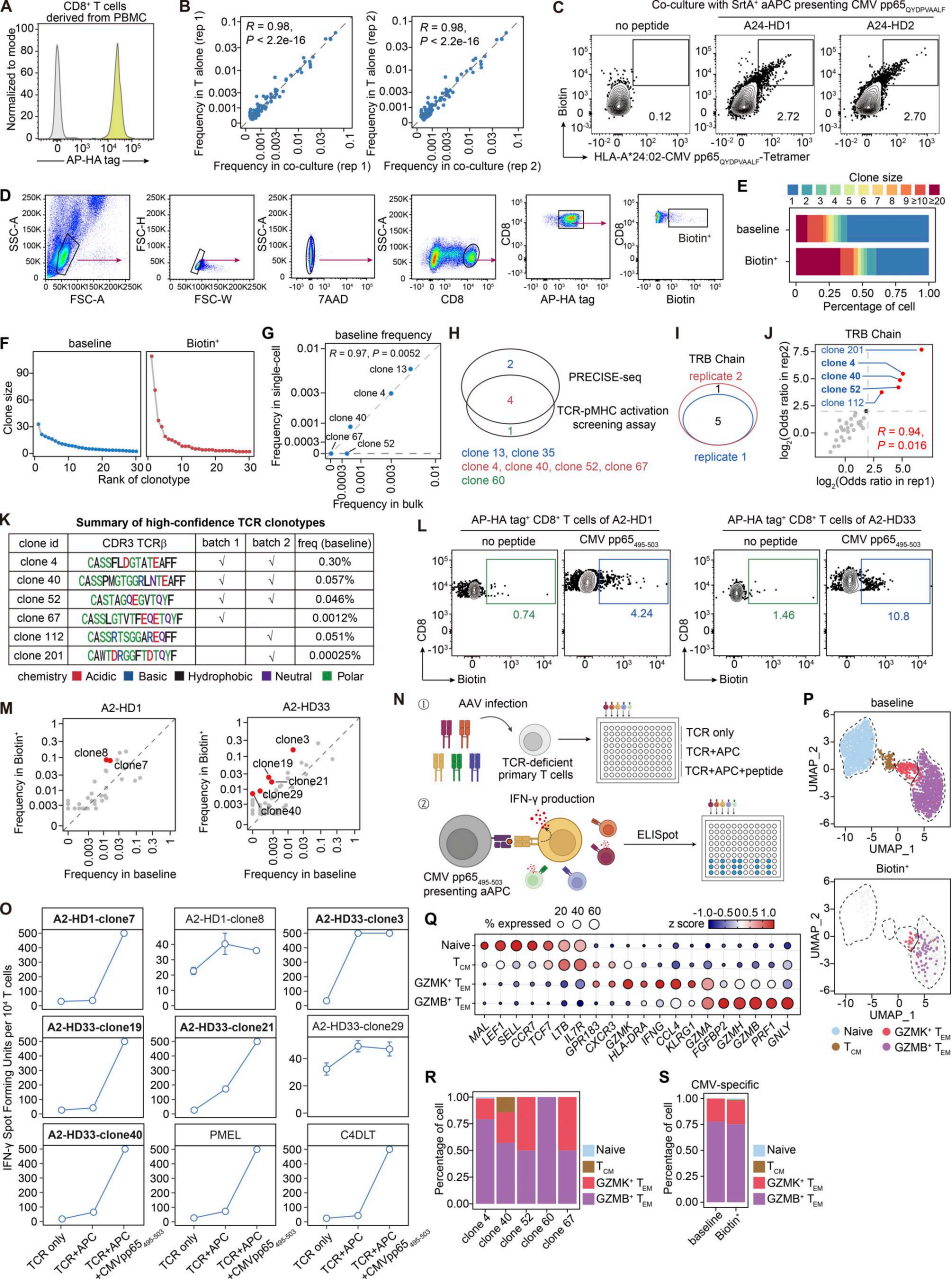
**TCR repertoire and transcriptional profile of human circulating CMV-specific T cells. (A)** Histograms showing the abundance of the AP-HA tag on human CD8^+^ T cells derived from PBMCs. **(B)** Comparison of T cell clone frequencies before and after 2-h coculture with K562-HLA-A2 cells presenting CMV pp65_495–503_. Data are presented as two technical replicates (*n* = 2). The frequency of each clonotype was determined by the detected TCRβ frequency in bulk TCR-seq. Pearson’s correlation was shown. **(C)** Flow cytometry analysis of Biotin and HLA-A*24:02-CMV pp65_QYDPVAALF_-Tetramer-PE staining on AP-HA tag^+^ CD8^+^ T cells from PBMCs of two HLA-A*24:02^**+**^ donors in the assay for identification of T cells specific to the CMV pp65_QYDPVAALF_ epitope. AP-HA tag^+^ CD8^+^ T cells cocultured with mock-pulsed SrtA^+^ aAPCs served as the negative control to determine the Biotin^+^ population for PRECISE-seq. **(D)** Gating strategy for defining the Biotin^+^ population (live CD8^+^AP-HA tag^+^Biotin^+^ single cells) of PRECISE-seq. CD8^+^ T cells with Biotin intensity exceeding 10^3^ were defined as the Biotin^+^ population and subsequently sorted for sequencing. **(E)** Bar chart depicting cell proportions of TCR clonotypes with varying clone sizes in PRECISE-seq. For a fair comparison, the baseline group was subsampled to match the number of cells in the Biotin^+^ group. The chart shows the aggregated results from subsampling the baseline group 10 times. **(F)** Clone size distribution of the top 30 clones in the baseline group and the Biotin^+^ group. Cells from the baseline group were subsampled to match the size of the Biotin^+^ group (10 times). The average clone size of the subsampled baseline groups was then calculated and presented. **(G)** Comparison of the baseline frequency of the high-confidence CMV-specific clonotypes, as determined by bulk TCR-seq (x axis) versus PRECISE-seq (y axis). **(H)** Venn diagram showing the number of significantly enriched clonotypes (FDR < 0.05 and OR > 4) identified by PRECISE-seq and the TCR-pMHC activation screening assay. **(I)** Venn diagram showing the number of significantly enriched clonotypes (FDR < 0.05 and OR > 4) identified in the two technical replicates of an independent PRECISE-seq experiment (batch 2). To ensure more sensitive detection, bulk TCR-seq was utilized. **(J)** Comparison of the ORs between two technical replicates for TCRβ clones used in the enrichment analysis (Biotin^+^ versus baseline). Significantly enriched clonotypes were defined as FDR < 0.05 and OR > 4. Pearson’s correlation was shown. **(K)** Summary table of high-confidence CMV-specific TCR clonotypes identified by the two batches of PRECISE-seq. **(L)** Flow cytometry analysis of Biotin signals on AP-HA tag^+^ CD8^+^ T cells from PBMCs of donors HLA-A2^+^ HD1 (left) and HLA-A2^+^ HD33 (right) in the assay for identification of T cells specific to the CMV pp65_495–503_ epitope. AP-HA tag^+^ CD8^+^ T cells cocultured with mock-pulsed SrtA^+^ aAPC served as the negative control to determine the Biotin^+^ population for PRECISE-seq. **(M)** Frequencies of TCR clonotypes in the baseline (x axis) group and the Biotin^+^ group (y axis) in PRECISE-seq of donors HLA-A2^+^ HD1 (left) and HLA-A2^+^ HD33 (right). High-confidence CMV-specific clonotypes (red) were defined by Fisher’s exact test (FDR < 0.05 and OR > 4). **(N)** Schematic illustration showing the antigen specificity validation of candidate T cell clonotypes identified by PRECISE-seq using the IFN-γ ELISpot assay. The candidate TCRs were synthesized and transfected into TCR-deficient primary T cells. The antigen specificity of TCR was then assessed by IFN-γ production under cognate CMV pp65_495–503_ stimulation. **(O)** Statistical analysis of candidate TCR-transfected primary T cells following in vitro coculture with K562-HLA-A2 cells presenting the CMV pp65_495–503_ peptide. T cells cultured alone or with target cells lacking the peptide served as negative controls. Positive controls comprised T cells expressing the reference TCRs (PMEL and C4DLT) cocultured with target cells loaded with their cognate peptides (HLA-A*02:01-restricted gp100_RMFPNAPYL_ and WT1_YLEPGPVTA_, respectively). **(P)** UMAP plot showing four clusters of CD8^+^ T cells derived from PBMCs. Cells from the baseline group (top) and the Biotin^+^ group (bottom) were shown. The low-confidence CMV-specific clones are shown in gray. **(Q)** Z score of the average expression of marker genes across each CD8^+^ T cell subset. **(R)** Phenotypic distribution of each CMV-specific clonotype in the Biotin^+^ group of PRECISE-seq. **(S)** Phenotypic distribution of CMV-specific T cells in the baseline group and the Biotin^+^ group.

We then utilized PRECISE-seq to systematically analyze the in vivo T cell response against human CMV, a common herpesvirus that periodically reactivates and thus repeatedly stimulates the antigen-specific T cells (Griffiths and Reeves, 2021) (Fig. 3 A). For this purpose, we isolated peripheral blood mononuclear cells (PBMCs) from a CMV-seropositive donor carrying HLA-A2 and labeled them with the AP-HA tag (Fig. S2 A). To identify CMV-specific CD8^+^ T cells, we incubated these AP-HA tag^+^ PBMCs with SrtA^+^ HLA-A2^+^ K562 cells presenting CMV pp65_495–503_ (Wills et al., 1996). We compared the TCR repertoire before and after coculture and found no significant alteration, thus ruling out potential artifacts from the coculture process (Fig. S2 B). Flow cytometry analysis revealed ∼1% of CD8^+^ T cells labeled with Biotin signals (Fig. 3 B). In addition, we performed parallel tetramer staining for direct comparison of the labeling efficiency using PBMCs from two CMV-seropositive donors carrying HLA-A*24:02. Flow cytometry analysis showed strong concordance between Biotin labeling and tetramer staining, confirming the efficacy of the PRECISE-seq approach (Fig. S2 C). The Biotin^+^ CD8^+^ T cells from HLA-A2^+^ donors were isolated and subsequently subjected to 5′ single-cell RNA sequencing (scRNA-seq), coupled with TCR profiling (Fig. S2 D). Additionally, nonconjugated CD8^+^ T cells were also isolated for single-cell sequencing to characterize the baseline transcriptional profile and TCR repertoire of polyclonal T cells in fresh PBMCs (hereafter referred to as the baseline group).

From the PRECISE-seq data, we recovered 1,450 clonotypes for the baseline group and 345 clonotypes for the Biotin^+^ group, respectively. Notably, the top clones in the Biotin^+^ group exhibited larger clone size compared with those in the baseline group, suggesting a selective enrichment of antigen-experienced, highly expanded TCR clonotypes (Fig. S2, E and F). To identify high-confidence CMV-specific clonotypes, we applied stringent criteria: (1) a minimum cell number threshold of 2 in the Biotin^+^ group; (2) significant enrichment in the Biotin^+^ group compared with the baseline group, with a false discovery rate (FDR) of <0.05 by Fisher’s exact test; and (3) an odds ratio (OR) >4. This strategy yielded six high-confidence clonotypes in the Biotin^+^ group, namely, clones 4, 13, 35, 40, 52, and 67. Notably, three of these clonotypes (clones 35, 52, and 67) were undetectable at baseline, highlighting the sensitivity of PRECISE-seq in detecting low-frequency antigen-specific clonotypes that might be overlooked by conventional single-cell TCR-sequencing (scTCR-seq) methods (Fig. 3 C).

It is important to note that when analyzing samples with an extensive TCR repertoire such as PBMCs, the number of cells captured by single-cell sequencing is limited by the maximum throughput capacity (typically up to 10^4^ cells in the 10X droplet-based platform), potentially resulting in a representation that does not fully reflect the starting population. Consequently, antigen-specific clonotypes with a low frequency (<0.01%) cannot be accurately quantified. To achieve more precise quantification of baseline frequency, we conducted bulk TCRα/β chain repertoire sequencing on baseline CD8^+^ T cells (bulk TCR-seq). The baseline frequencies of high-confidence clonotypes detected in bulk TCR-seq and PRECISE-seq were highly correlated (Fig. S2 G). However, bulk TCR-seq enabled the detection of clones 52 and 67 with baseline frequencies as low as 0.046% and 0.0012%, respectively. Notably, these two clonotypes were identified as high-confidence clonotypes by PRECISE-seq, demonstrating the ability of our method to identify extremely low-frequency antigen-specific T cells (Fig. 3 D).

Next, we evaluated the accuracy of PRECISE-seq using a TCR-pMHC activation screening assay. We selected 245 clonotypes to generate an adeno-associated virus (AAV) vector-based TCR library, which contained: (1) six high-confidence clonotypes and 221 low-confidence clonotypes identified by PRECISE-seq in the Biotin^+^ group; (2) 18 clonotypes only detected in the baseline group. In addition, three reported CMV pp65_495–503_-specific clonotypes as positive controls (Shugay et al., 2018). Subsequently, we transduced this TCRαβ expression library into TCR-deficient human primary T cells. We performed the activation screening assay in the presence of CMV pp65_495–503_ peptide and then sorted activated cells (4-1BB^+^) to identify CMV-specific clonotypes (Gros et al., 2016) (Fig. 3 E). The CMV-specific TCR clonotypes were defined as those enriched in the CMV pp65_495–503_-stimulated group compared with control groups (Materials and methods). While three reported TCRs were enriched in the activation screening assay, four of six high-confidence clonotypes (clones 4, 40, 52, and 67) identified by PRECISE-seq were also enriched in this screening assay (Fig. 3 F and Fig. S2 H). Only one out of 221 low-confidence clonotypes (clone 60) was enriched. None from the baseline group were enriched in the screening assay. Additionally, three out of four true-positive CMV-specific clones were reproducibly identified in an independent experimental batch of PRECISE-seq (Fig. S2, I–K). Moreover, applying PRECISE-seq to two additional CMV-seropositive donors successfully retrieved their respective CMV pp65_495–503_-specific clonotypes, as validated by IFN-γ ELISpot assay (Fig. S2, L–O). Overall, these results indicate that PRECISE-seq effectively and accurately retrieves antigen-specific TCRs from a large pool of TCR repertoire in circulating blood upon CMV infection.

To quantify the potency of TCR-pMHC interactions for individual clonotypes, we assigned a potency score to each clonotype by calculating the fold change in frequency between the Biotin^+^ group and the baseline group (determined by bulk TCR-seq) and then adjusting it based on the distribution of irrelevant TCRs (Materials and methods). Among the four CMV-specific clonotypes, clone 4 was classified as high-potency, and clones 40 and 52 as intermediate-potency, and clone 67 was excluded from the analysis due to its extremely low baseline frequency (0.0012%), as such a small denominator makes the fold-change estimate highly susceptible to stochastic noise (Fig. 3 D). We then evaluated whether the potency scores obtained through PRECISE-seq correlate with the in vitro TCR avidity assessed by flow cytometry. To this end, JC5 cells were transduced with CMV-specific TCRs and stimulated with serially diluted CMV pp65_495–503_ peptide presented by K562-HLA-A2 cells. TCR avidity was quantified by flow cytometry for CD69 staining, measuring the peptide concentration required for a half-maximal response. All TCR clones demonstrated peptide dose-dependent CD69 upregulation, and the measured avidity (EC_50_) was consistent with the potency scores assessed by PRECISE-seq, maintaining a similar ranking among the clones (Fig. 3 G). We noticed that the functional avidity of clone 67 was 220-fold lower than clone 4, whose potency was beyond the detection scope of PRECISE-seq. These results validate that the TCR potency scores derived from PRECISE-seq serve as a reliable proxy for functional avidity.

### High-potency CMV-specific T cells acquired an exhausted phenotype

**Figure 4.**
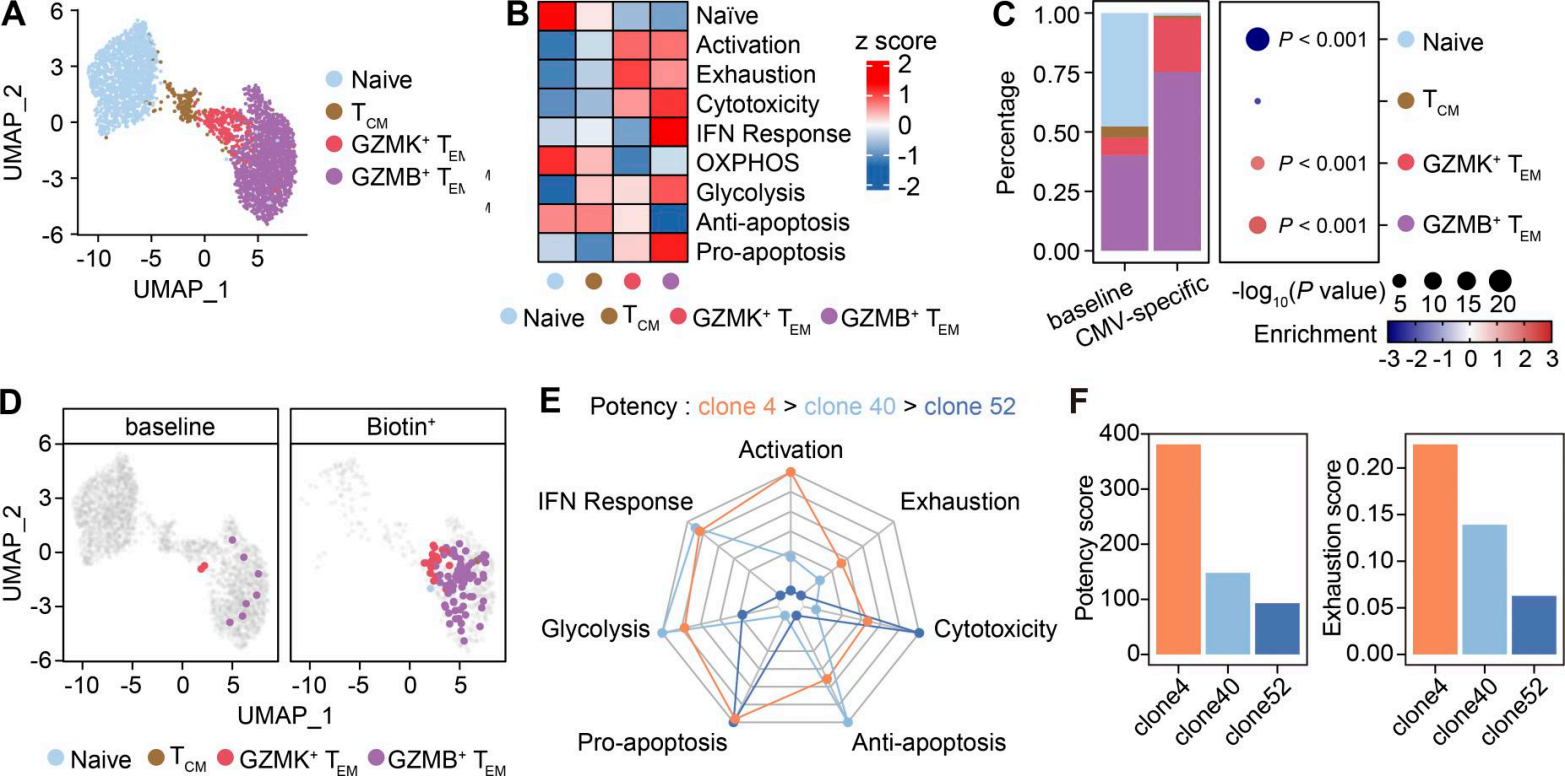
**High-potency CMV-specific clonotypes tend to adopt an exhaustion phenotype. (A)** UMAP plot showing four clusters of CD8^+^ T cells derived from PBMCs. Cells from the baseline group and the Biotin^+^ group were shown together. **(B)** Heatmap showing the z score for the average expression of gene signatures in each CD8^+^ T cell subset. IFN, interferon; OXPHOS, oxidative phosphorylation. **(C)** Phenotypic distribution (left) and enrichment analysis (right) of CMV-specific clonotypes in the Biotin^+^ group compared with all clonotypes in the baseline group. The P value was determined using Fisher’s exact test. **(D)** UMAP plot showing the phenotype of CMV-specific TCR clonotypes in the baseline group and Biotin^+^ group of PRECISE-seq. **(E)** Radar chart showing the z score for the average expression of gene signatures in each CMV-specific TCR clonotype. Only effector memory cells were included in the analysis. **(F)** Bar plot illustrating the correlation between potency scores and the expression of exhaustion signature across CMV-specific TCR clonotypes.

We utilized PRECISE-seq to explore the phenotypic landscape of CMV-specific T cells through analysis of transcriptome data. Consistent with previous encounters with the antigen, CMV-specific clonotypes were notably devoid of naïve cells, which constitute a major population in the baseline group (Fig. 4 A and Fig. S2 P). Interestingly, CMV-specific T cells were predominantly found within two effector memory T cell (T_EM_) subsets, characterized by high levels of activation and cytotoxicity signatures (GZMB^+^ T_EM_ and GZMK^+^ T_EM_) (Fig. 4, B–D; and Fig. S2, Q and R). We verified that the effector memory phenotype of CMV-specific clonotypes was not induced by the labeling process of PRECISE-seq, as these clonotypes exhibited a similar phenotypic distribution in the baseline group (Fig. S2 S). Considering that TCR potency determines the in vivo functional phenotype of antigen-specific T cells (Oliveira et al., 2021; Schmidt et al., 2023), we examined the correlation between TCR potencies and T cell phenotypes. We observed that high-potency T cells exhibited increased expression of an activation signature, suggesting a greater magnitude of T cell activation resulting from enhanced APC-T intercellular interactions (Fig. 4 E). Notably, high-potency T cells also upregulated the expression of an exhaustion signature, consistent with the prior notion that iterative antigen stimulation drives T cell exhaustion program (Klenerman and Oxenius, 2016) (Fig. 4 F). Overall, PRECISE-seq effectively delineates the antigen specificity, TCR potency, and phenotypic landscape of CMV-specific clonotypes, enabling comprehensive characterization of T cell responses to human CMV infection.

### Functional profiling of tumor-specific T cells in the tumor microenvironment (TME)

**Figure S3.**
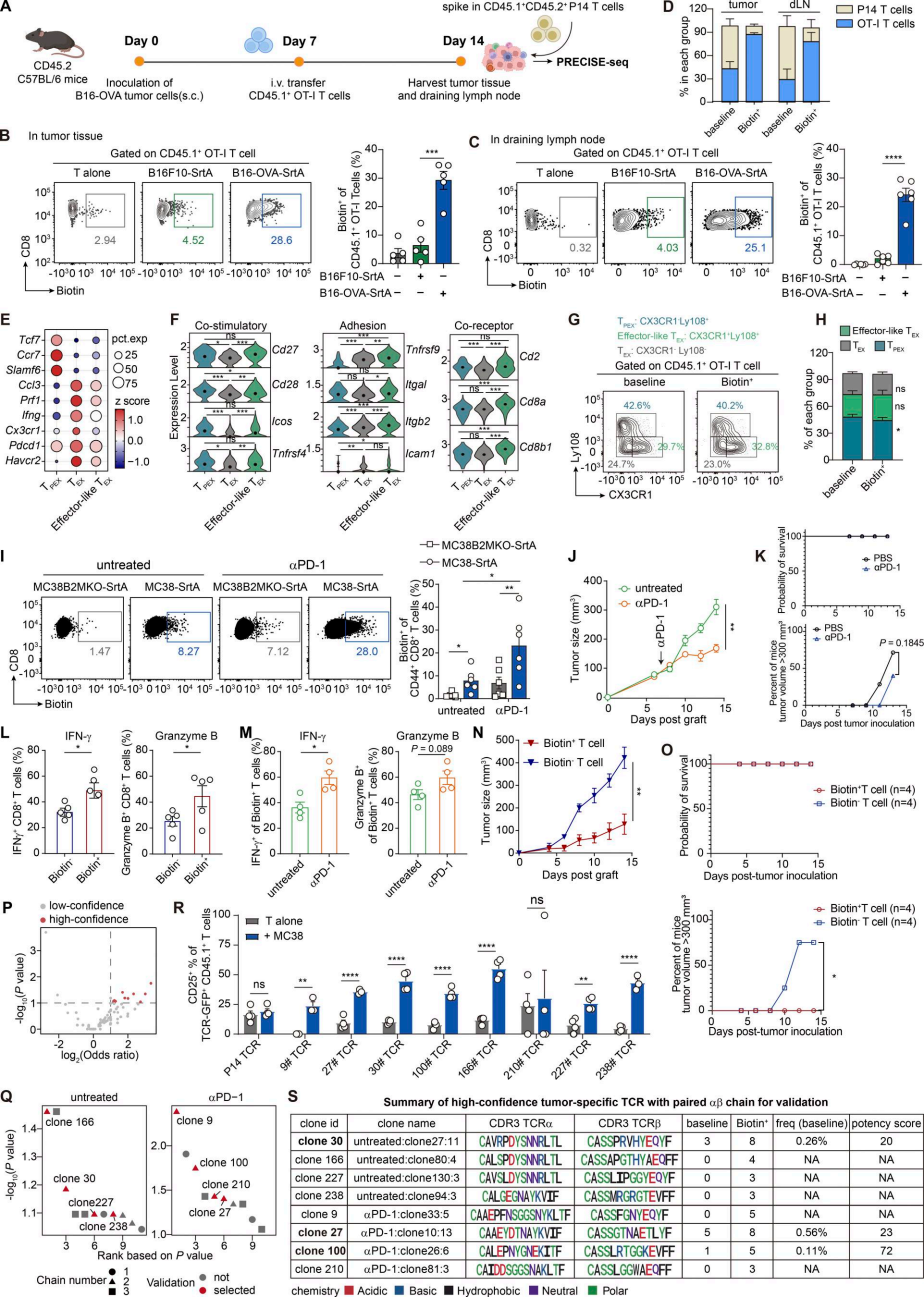
**PRECISE-seq for monitoring of in vivo antitumor T cell responsiveness. (A)** Schematic view of PRECISE-seq for identifying tumor antigen-specific T cells from the B16-OVA tumor model. CD45.2 C57BL/6 mice were subcutaneously inoculated with B16-OVA tumor cells (5 × 10^5^) and received i.v. adoptive transfer of naïve CD45.1^+^ OT-I T cells (5 × 10^5^) at day 7 after tumor inoculation. Tumor tissues and tuDLNs were harvested and enzymatically digested into single-cell suspension on day 14. Antigen-irrelevant CD45.1^+^ CD45.2^+^ P14 T cells isolated from the spleen were spiked into the single-cell suspension to achieve a final abundance comparable to that of the OT-I T cells. After conjugation of the AP-HA tag, the total population of CD8^+^ T cells was cocultured with B16-OVA-SrtA tumor cells and analyzed by flow cytometry. **(B)** Representative flow plots (left) and statistical analysis (right) showing the frequencies of the Biotin^+^ cells within tumor-infiltrating CD45.1^+^ OT-I T cells labeled by the PRECISE strategy. **(C)** Representative flow plots (left) and statistical analysis (right) showing the frequencies of the Biotin^+^ cells within CD45.1^+^ OT-I T cells derived from draining lymph nodes. **(D)** Evaluation of the relative abundance of antigen-specific OT-I T cells (positive control) and bystander P14 T cells (negative control) in the baseline (total nonconjugated CD8^+^ T cells) and the Biotin^+^ group. **(E)** z score of the average expression of marker genes across three CD8^+^ T cell subsets in a public scRNA-seq dataset of B16 melanoma (GSE116390). **(F)** Violin plots showing the expression profiles of costimulatory molecules, adhesion molecules, and coreceptors among various T cell subsets. The P values were determined by a two-sided Wilcoxon rank-sum test. ns represents P > 0.05; * represents P < 0.05; ** represents P < 0.01; *** represents P < 0.001. **(G)** Representative flow plots depicting the frequencies of T_PEX_ (Ly108^+^ T_PEX_ cells), effector-like T_EX_ (PD-1^+^ CX3CR1^+^ Ly108^−^ effector-like T_EX_ cells), and terminal T_EX_ (PD-1^+^ Tim-3^+^ Ly108^−^ CX3CR1^−^ T_EX_ cells) across both the baseline and the Biotin^+^ groups. **(H)** Statistical analysis of G. **(I)** Representative flow plots (left) and statistical analysis (right) showing the frequencies of the Biotin^+^ cells in tumor-infiltrating CD8^+^ T cells from untreated and αPD-1 Ab–treated MC38 tumor tissues. The MC38 tumor-bearing mice were randomly grouped based on tumor size and treated with 200 μg αPD-1 Ab on day 7 after tumor inoculation. **(J)** Tumor growth of MC38 tumor cells under αPD-1 Ab treatment. C57BL/6 mice were s.c. inoculated with MC38 tumor cells (5 × 10^5^), and i.p. injected with 200 μg αPD-1 Ab on day 7; *n* = 5–7 in each group. **(K)** Survival curve of untreated or αPD-1 Ab–treated MC38 tumor-bearing mice in J. **(L)** Bar plots showing the cytokine production of Biotin^+^ and Biotin^−^ tumor-infiltrating CD8^+^ T cells derived from untreated MC38 tumor tissues at day 14 after tumor inoculation. **(M)** Quantitative analysis of cytokine production of tumor antigen-specific (Biotin^+^) CD8^+^ T cells from MC38 tumor-bearing mice with or without αPD-1 Ab treatment. **(N)** Evaluation of the antitumor capacity of Biotin^+^ and Biotin^−^ tumor-infiltrating CD8^+^ T cells in vivo. 1,000 Biotin^+^/Biotin^−^ CD45.1^+^ CD8^+^ T cells were i.v. transferred into Rag2^−/−^ mice, followed by s.c. inoculation of 3 × 10^5^ MC38 cells. The tumor curves were measured starting on day 6 and every 2 days following that. **(O)** Survival curve of MC38 tumor-bearing mice received Biotin^+^ T cell or Biotin^−^ T cell adoptive transferring therapies in N. **(P)** Enrichment analysis for the identification of tumor-specific clonotypes. The high-confidence tumor-specific clonotypes (red) were defined as expanded clones (N > 1 in the Biotin^+^ group) with a P value <0.1 and an OR >2 in the Biotin^+^ group compared with the baseline group through Fisher’s exact test. **(Q)** Dot plots showing the P value rankings of high-confidence mouse tumor-specific TCRs. The TCR clonotypes selected for validating the antigen specificity are indicated in red. **(R)** Bar plot showing the expression of CD25 on TCR-transduced T cells following an 18-h coculture with MC38 tumor cells. Error bars represent the mean ± SEM. Each dot represents a different technical replicate; *n* = 3–4; statistical significance is determined by unpaired, one-tailed Student’s *t* test. ns represents P > 0.05; * represents P < 0.05; ** represents P < 0.01; **** represents P < 0.0001. **(S)** Summary table of high-confidence tumor-specific TCR clonotypes identified by PRECISE-seq. The TCR clonotypes selected for assessing the potency score are indicated in bold. The abbreviation NA signifies not available. Data are representative of three or four independent experiments and *n* = 4–6 independent animals, unless otherwise noted. Each dot represents an individual biological replicate. Data are presented as the mean ± SEM, and results were compared by unpaired, two-tailed Student’s *t* test. ns represents P > 0.05; * represents P < 0.05; ** represents P < 0.01; *** represents P < 0.001; **** represents P < 0.0001.

We applied PRECISE-seq to monitor the dynamics of in vivo antitumor T cell response occurring within the TME upon immunotherapy. Tumor-specific T cells experiencing chronic antigen exposure undergo TCR internalization, which reduces the availability of TCRs for binding with pMHC and limits the detection of tumor-specific T cells within the TME (Stinchcombe et al., 2023). Initially, we confirmed the ability of PRECISE-seq to capture tumor-specific T cells within tumor tissues and tumor-draining lymph nodes (tuDLNs) by transferring OT-I T cells into mice bearing B16-OVA tumors (Fig. S3 A). OT-I T cells from tumor or tuDLNs were explicitly labeled with Biotin signals by B16-OVA-SrtA, while negligible Biotin signals were detected after coculturing with B16F10-SrtA (Fig. S3, B and C). Moreover, the irrelevant P14 T cells, which are reactive to the LCMVgp_33–41_ epitope and were spiked into tumor-infiltrating cells as an internal negative control, were also Biotin-negative (Fig. S3 D), reinforcing the high specificity of PRECISE-seq in detecting T cells against tumor antigens. In addition, the Biotin labeling intensity was comparable among different T cell subsets, despite their varying expression of adhesion molecules and costimulation molecules (Fig. S3, E–H), indicating that PRECISE-seq is not confounded by differences in T cell phenotypes.

**Figure 5.**
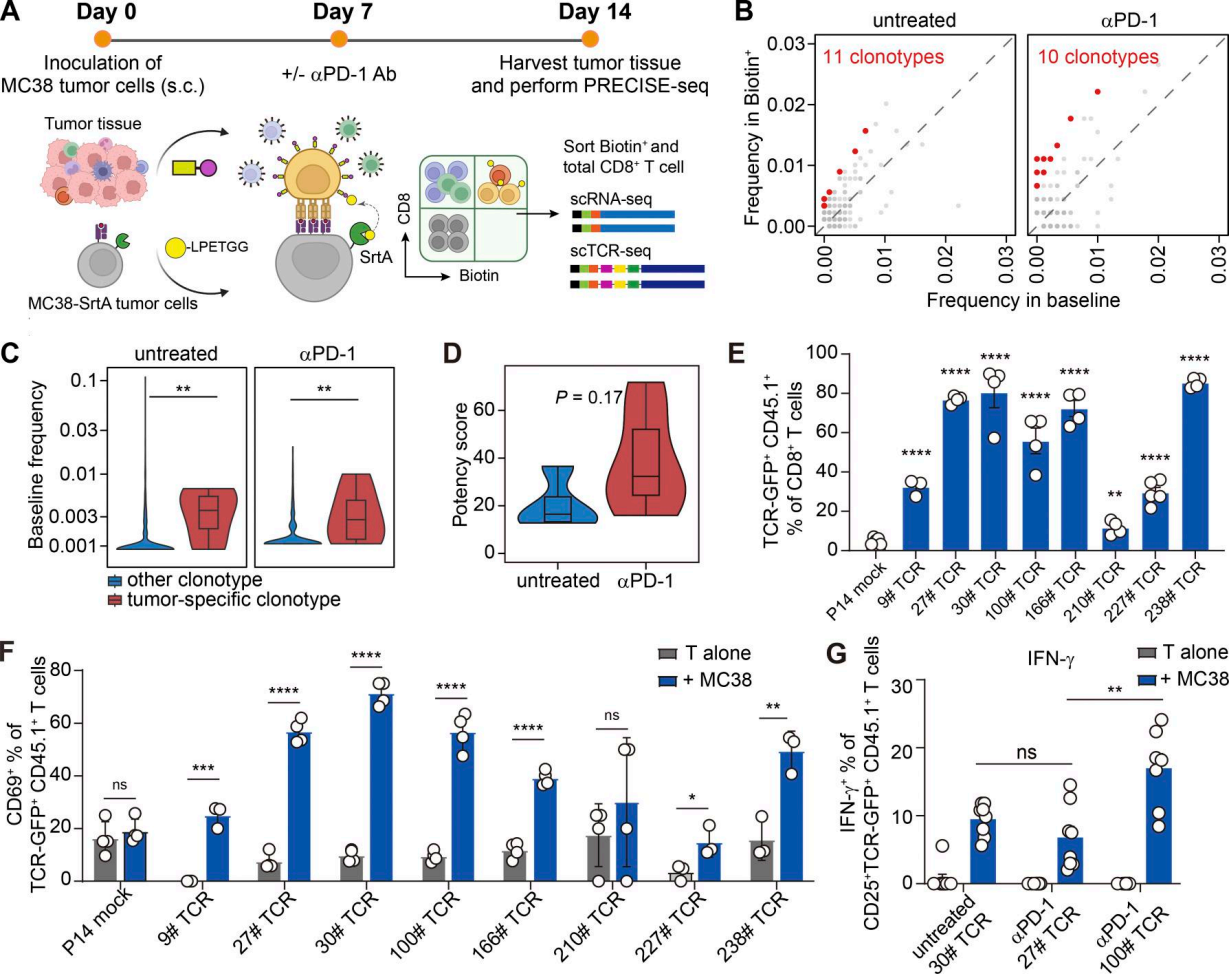
**PRECISE-seq enables rapid identification of tumor-specific T cells from MC38 tumor model under αPD-1 treatment. (A)** Schematic overview of PRECISE-seq for characterizing antigen specificity and monitoring phenotype dynamics of tumor-infiltrating T cells from the MC38 tumor model under immunotherapy. MC38 tumor-bearing mice were treated with 200 μg αPD-1 Ab on day 7. CD8^+^ T cells were harvested from tumor tissues on day 14 after tumor inoculation and cocultured with MC38-SrtA tumor cells. PRECISE-seq was conducted after 2 h of coculture. **(B)** Dot plot showing the frequencies of CD8^+^ T cells in the Biotin^+^ (y axis) and the baseline group (x axis). High-confidence tumor-specific clonotypes (red) were defined as expanded clones (N > 1 in the Biotin^+^ group) with a P value <0.1 and an OR >2 in the Biotin^+^ group compared with the baseline group through Fisher’s exact test. **(C)** Frequencies of high-confidence tumor-specific clonotypes and other clonotypes in the baseline group. The P value was determined by a two-sided Wilcoxon rank-sum test. ** represents P < 0.01. **(D)** Violin plot showing the potency scores of tumor-specific clonotypes in untreated tumors and tumors treated with αPD-1 Ab. Only clonotypes with detected baseline frequencies were included in the calculation of the potency score. **(E)** Bar plot showing the frequencies of TCR-GFP^+^ T cells among total CD8^+^ T cells in tumor tissues. Tumor-infiltrating CD8^+^ T cells were harvested 15 days after tumor inoculation, and the clonal expansion of transferred TCR-GFP^+^ T cells was assessed by flow cytometry. Error bars represent the mean ± SEM. Statistical significance is determined by unpaired, two-tailed Student’s *t* test; ** represents P < 0.01; **** represents P < 0.0001. **(F)** Bar plot showing the expression of CD69 on TCR-transduced T cells following an 18-h coculture with MC38 tumor cells. Error bars represent the mean ± SEM. Statistical significance is determined by unpaired, one-tailed Student’s *t* test; ns represents P > 0.05; * represents P < 0.05; ** represents P < 0.01; *** represents P < 0.001; **** represents P < 0.0001. **(G)** Bar plot showing the IFN-γ secretion of CD25^+^ TCR-transduced T cells following an 18-h coculture with MC38 tumor cells. Error bars represent the mean ± SEM. Each dot represents a different technical replicate; *n* = 8 per group; statistical significance is determined by unpaired, two-tailed Student’s *t* test; ns represents P > 0.05; ** represents P < 0.01.

Understanding the underlying mechanisms of productive antitumor T cell responses upon immunotherapy requires accurate characterization of alterations in both T cell phenotype and TCR repertoire. We hypothesized that PRECISE-seq could elucidate the in vivo antitumor T cell responsiveness by linking dynamic T cell landscapes and TCR specificities against the available antigens presented by tumor cells. To investigate this, we implanted mouse colon tumor cells MC38 into C57BL/6 mice and treated tumor-bearing mice with PD-1 blockade antibody (αPD-1 Ab) (Fig. 5 A). Tumor antigens were presented by an autologous MC38 tumor cell line (Yadav et al., 2014), with SrtA overexpressed on the surface of MC38 tumor cells. 7 days after treatment, we cocultured AP-conjugated tumor-infiltrating cells with either MC38-SrtA cells or MHCI-deficient MC38-SrtA cells (MC38 B2MKO-SrtA cells) and then sorted the Biotin^+^ CD8^+^ T cells to perform PRECISE-seq. Additionally, we isolated total tumor-infiltrating CD8^+^ T cells without AP-HA tag conjugation to establish a reference for the TCR repertoires within tumors (baseline).

We observed a notable increase in the frequency of Biotin-labeled CD8^+^ T cells in mice treated with αPD-1 Ab compared with those in untreated tumors (Fig. S3 I), correlating with a significant suppression of tumor growth under αPD-1 Ab treatment (Fig. S3, J and K). Flow cytometry analysis revealed that Biotin^+^ tumor-infiltrating CD8^+^ T cells exhibited heightened levels of effector cytokine production compared with their Biotin^−^ counterparts (Fig. S3 L). Additionally, PD-1 blockade further augmented the production of effector cytokines in Biotin^+^ CD8^+^ T cells, underscoring the role of PD-1 blockade in enhancing tumor-specific T cell responses (Fig. S3 M). Furthermore, the transfer of Biotin^+^ CD8^+^ T cells into MC38 tumor-bearing mice resulted in inhibited tumor growth, whereas tumors progressed in mice receiving Biotin^−^ CD8^+^ T cells (Fig. S3, N and O), confirming the functional responsiveness of Biotin-labeled CD8^+^ T cells to tumor antigens.

When analyzing the PRECISE-seq data, we identified 1,220 CD8^+^ T cell clonotypes in the baseline group and 719 clonotypes in the Biotin^+^ group, among which 21 were designated as high-confidence tumor-specific clonotypes (Fig. 5 B and Fig. S3 P). These tumor-specific clonotypes displayed higher baseline frequencies compared with other clonotypes, indicating a continuous expansion of tumor-specific T cells in response to tumor antigens within the TME (Fig. 5 C). Subsequently, we computed potency scores for the high-confidence tumor-specific clonotypes. Compared with CMV-specific clonotypes, tumor-specific clonotypes with available potency scores exhibited significantly lower potency (P = 0.006, two-sided Wilcoxon rank-sum test). This result supports the concept that natural T cell repertoires targeting tumor antigens tend to express TCRs with relatively low potency (Klein et al., 2014; Nikolich-Zugich et al., 2004). Following αPD-1 treatment, a modest increase in TCR potency was observed, suggesting that the increase in TCR potency may contribute to the antitumor effects mediated by αPD-1 therapy (Fig. 5 D).

To validate the accuracy of PRECISE-seq in retrieving tumor-specific clonotypes, we selected eight dominant TCRs based on their P value rankings from both the untreated and αPD-1 groups and examined their tumor reactivity (Fig. S3 Q). We transduced tumor-irrelevant P14 T cells with retroviral constructs encoding the TCR chains of these clonotypes and transferred these TCR-transduced T cells into MC38 tumor-bearing mice, using GFP-transduced P14 T cells as a tumor-irrelevant negative control. We found that the cell proportions of all eight clonotypes were significantly higher than those of the control P14 T cells in tumors 7 days after transfer (Fig. 5 E), suggesting that these TCR-transduced T cells underwent robust proliferation upon recognizing antigens on tumor cells. To confirm tumor reactivity, we re-stimulated tumor-infiltrating lymphocytes (TILs) ex vivo with MC38 cells and evaluated the reactivity by the upregulation of the TCR downstream signaling markers CD69 and CD25. Seven out of eight clones exhibited significant induction of CD69 and CD25 upon re-exposure to MC38 cells, confirming their tumor reactivity (Fig. 5 F and Fig. S3 R). To determine whether TCR potency assessed by PRECISE-seq correlates with the ex vivo functional avidity of tumor-specific TCR clonotypes, three clonotypes were selected for downstream analysis: clone 100 (high TCR potency) and clones 27 and 30 (low TCR potency), as measured by PRECISE-seq (Fig. S3 S). We compared the functional response of these three clonotypes by evaluating their capacity to produce IFN-γ. Flow cytometry analysis revealed that clone 100 exhibited significantly higher IFN-γ production than clones 27 and 30 (Fig. 5 G). This consistency with the TCR potency scores from PRECISE-seq confirms its effectiveness as a surrogate for assessing the functionality of tumor-specific T cells.

### Attenuated tumor-specific T_Ly49_ formation as a mode of action for αPD-1 therapy

**Figure 6.**
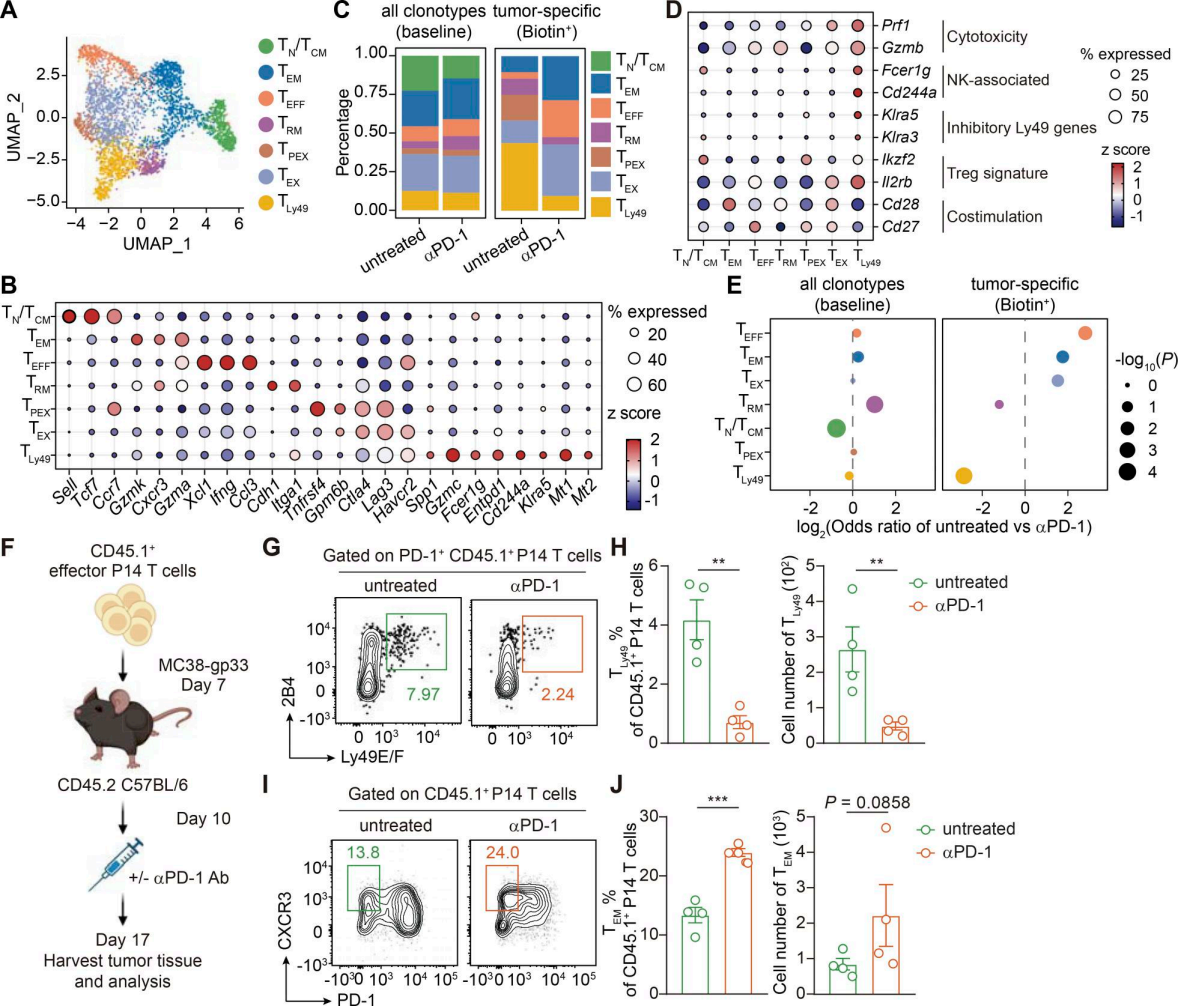
**Phenotypic plasticity of tumor-specific T cells responding to PD-1 blockade. (A)** UMAP plot displaying seven subsets of CD8^+^ T cells detected via PRECISE-seq. Cells from the Biotin^+^ and the baseline groups are shown together. **(B)** z score of the average expression of marker genes across each CD8^+^ T cell subset. **(C)** Bar chart displaying the phenotypic distribution of all clonotypes in the baseline group (left) and high-confidence tumor-specific clonotypes in the Biotin^+^ group (right). **(D)** Dot plot displaying transcript levels of selected signature genes of T_Ly49_ cells in scRNA-seq data of the MC38 tumor model. **(E)** Bar chart displaying the phenotypic distribution of all clonotypes in the baseline group (left) and high-confidence tumor-specific clonotypes in the Biotin^+^ group (right). T_PEX_ cells in the Biotin^+^ group exhibited a negative infinity OR, which was not shown. **(F)** Experimental design for phenotypic profiling of tumor-specific T cells under αPD-1 Ab treatment. CD45.2 C57BL/6 mice were subcutaneously inoculated with MC38-gp33 tumor cells (5 × 10^5^) and received i.v. adoptive transfer of activated CD45.1^+^ P14 T cells (2 × 10^5^) at day 7 after tumor inoculation. The MC38-gp33 tumor-bearing mice were then randomly grouped based on tumor curves (*n* = 4–6 per group). For the αPD-1 treatment group, 200 μg αPD-1 Ab was i.p. injected into tumor-bearing mice on day 10 after tumor inoculation. Tumor-infiltrating T cells were harvested on day 17 and subjected to phenotypic profiling by flow cytometry. **(G)** Representative flow plots illustrating the frequencies of T_Ly49_ (PD-1^+^2B4^+^Ly49E/F^+^) among tumor-infiltrating PD-1^+^ CD45.1^+^ P14 T cells in untreated or αPD-1–treated groups. **(H)** Bar plot showing the frequencies and absolute numbers of T_Ly49_ within MC38-gp33 tumors in untreated or αPD-1–treated groups. **(I)** Representative flow plots illustrating the frequencies of T_EM_ (PD-1^−^ CXCR3^+^) among tumor-infiltrating CD45.1^+^ P14 T cells in untreated or αPD-1–treated groups. **(J)** Bar plot showing the frequencies and absolute numbers of T_EM_ within MC38-gp33 tumors in untreated or αPD-1–treated groups. Data (F–J) are representative of three or four independent experiments and *n* = 4–6 independent animals unless otherwise noted. Each dot represents an individual biological replicate. Data are presented as the mean ± SEM. Statistical significance is determined by unpaired, two-tailed Student’s *t* test; ns represents P > 0.05; ** represents P < 0.01; *** represents P < 0.001.

**Figure S4.**
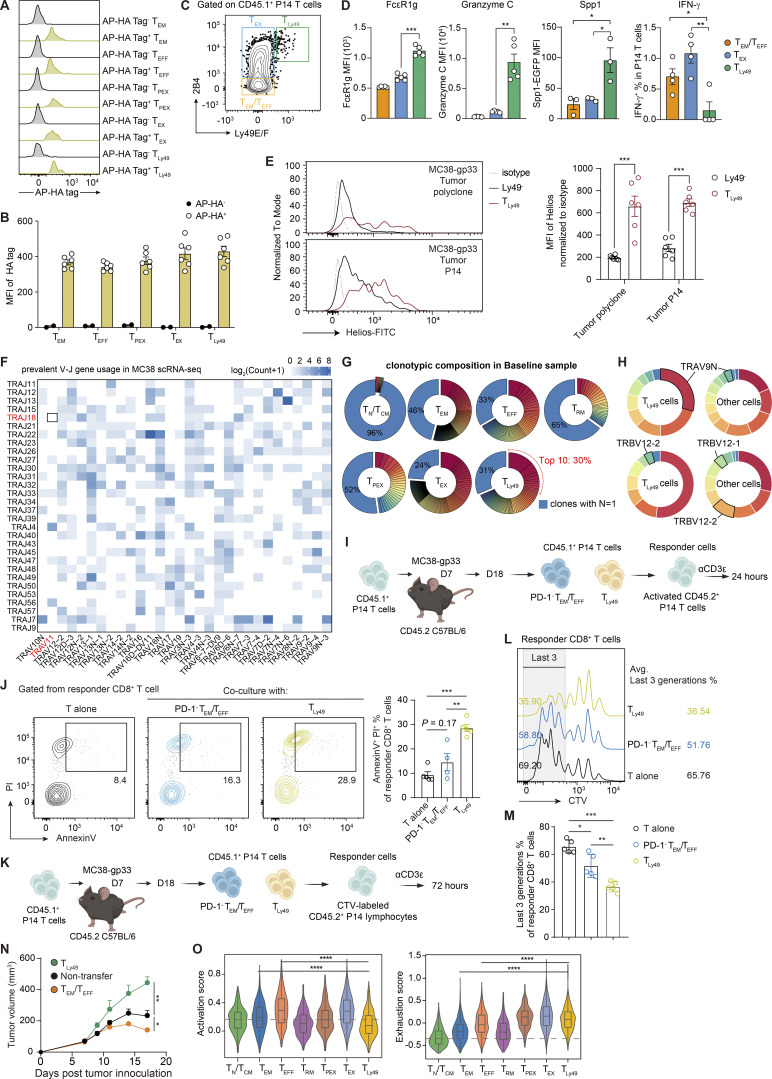
**Phenotypic and clonotypic comparison of tumor-specific T**_**Ly49**_ **cells and other T cell populations. (A)** Histograms showing the abundance of the AP-HA tag on tumor-infiltrating CD8^+^ T cell including different T cell subsets. CD45.2 C57BL/6 mice were subcutaneously inoculated with MC38-gp33 tumor cells. At day 21 after tumor inoculation, tumor-infiltrating CD45.2^+^ CD8^+^ T cells were harvested and conjugated with the AP-HA tag. The abundance of the AP-HA tag on tumor-infiltrating T cells was assessed by flow cytometry. Five T cell subsets were defined based on surface marker expression: T_EM_ (CXCR3^+^PD-1^+^), T_EFF_ (LAG3^−^), T_PEX_ (PD-1^+^Tim3^−^Ly108^+^), T_EX_ (PD-1^+^Tim3^+^Ly108^−^CD39^+^), and T_Ly49_ (2B4^+^Ly49E/F^+^). **(B)** Bar plot showing the statistical results of AP-HA tag levels across different T cell populations. Error bars represent the mean ± SEM. Each dot represents a different technical replicate; *n* = 6 per group. **(C)** Gating strategy for defining T_Ly49_, T_EX_, and T_EM_/T_EFF_ populations based on the expression of 2B4 and Ly49E/F. **(D)** Bar plots showing the expression of FcεR1g, granzyme C, SPP1, and IFN-γ on T_Ly49_, T_EX_, and T_EM_/T_EFF_ within tumor-infiltrating CD45.1^+^ P14 T cells derived from the untreated group. Data are representative of *n* = 3–5 independent animals. Each dot represents an individual biological replicate. Data are presented as the mean ± SEM. Statistical significance is determined by unpaired, two-tailed Student’s *t* test; * represents P < 0.05; ** represents P < 0.01; *** represents P < 0.001. **(E)** Histogram overlay and the MFI showing Helios expression in tumor-infiltrating Ly49^−^ CD8^+^ T cells and T_Ly49_ cells isolated from MC38-gp33 at day 17. Data (E) are representative of *n* = 6 independent animals. Each dot represents an individual biological replicate. Data are presented as the mean ± SEM. Statistical significance is determined by unpaired, two-tailed Student’s *t* test; *** represents P < 0.001. **(F)** Heatmap showing prevalent TCR variable gene usage of tumor-infiltrating T cells in the MC38 tumor model. **(G)** Clonotypic composition of various CD8^+^ T cell subsets in the baseline sample. **(H)** TCR variable gene usage in Biotin^+^ tumor-specific T cells. **(I and J)** CD45.2 C57BL/6 mice were inoculated subcutaneously with MC38-gp33 tumor cells (5 × 10^5^) and received i.v. adoptive transfer of activated CD45.1^+^ P14 T cells (2 × 10^5^) on day 7. On day 18, tumor-infiltrating T_EM_/T_EFF_ (PD-1^−^) and T_Ly49_ (PD-1^+^CXCR3^−^Ly49E/F^+^) P14 T cells were sorted and cocultured with preactivated CD45.2^+^ T_EFF_ for 24 h (I). Percentage of annexin V^+^/PI^+^ non-viable responder CD8^+^ P14 T cells after 24-h coculture (J). Data are representative of *n* = 4–5 independent replicates. Each dot represents an individual technical replicate. Data are presented as the mean ± SEM. Statistical significance is determined by unpaired, two-tailed Student’s *t* test; ** represents P < 0.01; *** represents P < 0.001. **(K–M)** Sorted T_EM_/T_EFF_ and T_Ly49_ P14 T cells were cocultured with CTV-labeled CD45.2^+^ lymphocytes for 72 h in the presence of 2 μg/ml anti-CD3 Ab stimulation. Representative histograms showing percentage of last three divisions of responder CD8^+^ T cells after 72-h coculture (K). The percentage of proliferating (last three division) responder CD8^+^ T cells (M). Data are representative of *n* = 5 independent replicates. Each dot represents an individual technical replicate. Data are presented as the mean ± SEM. Statistical significance is determined by unpaired, two-tailed Student’s *t* test; * represents P < 0.05; ** represents P < 0.01; *** represents P < 0.001. **(N)** Tumor growth curves depicting the progression of MC38-gp33 tumors following the adoptive transfer of T_EM_/T_EFF_ (LAG3^−^) or T_Ly49_ (LAG3^+^ 2B4^+^ Ly49E/F^+^), compared with a PBS control injection. Two T cell subsets, namely, T_EM_/T_EFF_ (LAG3^−^) and T_Ly49_ (LAG3^+^ 2B4^+^ Ly49E/F^+^), were isolated from tumor tissues using FACS and subsequently intratumorally injected into MC38-gp33 tumor-bearing mice 7 days after tumor inoculation. The tumor curves of recipient mice were measured starting 5 days after tumor inoculation and every 2 days following that. Data are representative of three or four independent experiments and *n* = 3 independent animals. Data are presented as the mean ± SEM, and results were compared by unpaired, two-tailed Student’s *t* test. * represents P < 0.05; ** represents P < 0.01. **(O)** Expression of the activation signature and exhaustion signature across CD8^+^ T cell subsets. The P value was determined by a two-sided Wilcoxon rank-sum test. **** represents P < 0.0001. MFI, median fluorescence intensity; CTV, CellTrace Violet.

To elucidate the phenotypic dynamics of polyclonal tumor-specific T cells following immune intervention, we conducted an analysis of the transcriptome data, partitioning all CD8^+^ T cells into seven subsets: T_N_ or central memory T cells (T_CM_), T_EM_, T_EFF_, tissue-resident memory cells (T_RM_), precursor of exhausted T cells (T_PEX_), exhausted T cells (T_EX_), and T_Ly49_ (Fig. 6, A and B). The comparable abundance of the AP-HA tag across different T cell subsets rules out any potential bias introduced by the conjugation process (Fig. S4, A and B). In untreated tumors, Biotin^+^ tumor-specific clonotypes mainly resided within the T_EX_ and T_Ly49_ compartments (Fig. 6 C). We noticed that these T_Ly49_ cells specifically expressed natural killer (NK)-related molecules such as FcεRIg, 2B4 (encoded by *Cd244a*), and the Ly49 family of inhibitory killer cell lectin-like receptors (*Klra3* and *Klra5*), resembling a previously reported Ly49^+^ regulatory CD8^+^ T cell (Kim et al., 2015; Li et al., 2022) (Fig. 6 D). In addition, T_Ly49_ from tumors shared many similarities with Ly49^+^ CD8^+^ regulatory T cells (with KIR^+^ CD8^+^ T cells as their human counterparts) identified in experimental autoimmune encephalomyelitis mice, as well as in human autoimmune and infectious diseases: a marked upregulation of cytotoxic molecules (*Gzmc* and *Prf1*), a loss of costimulatory molecules (*Cd28* and *Cd27*), and the upregulation of *Ikzf2*, a transcriptional factor associated with regulatory functions in both CD4^+^ and CD8^+^ T cells (Kim et al., 2015). Flow cytometry analysis confirmed that T_Ly49_ CD8^+^ T cells expressed high levels of FcεR1g, granzyme C, Helios, and immunosuppressive molecule SPP1 (OPN), but rarely produced effector cytokine IFN-γ (Fig. S4, C–E). Notably, we did not identify any cells co-expressing Trav11 (Vα14) and Traj18 in our system, thereby excluding the inclusion of invariant NKT cells within the T_Ly49_ subset (Fig. S4 F).

Analysis of the TCR clonotype distributions across all CD8^+^ T cell subsets at baseline revealed that T_EFF_, T_EX_, and T_Ly49_ cells showed the highest degrees of clonal expansion compared with naïve and memory subsets (Fig. S4 G). Notably, T_Ly49_ displayed a diverse TCR repertoire, composed of multiple expanded clonotypes rather than domination by a few hyperexpanded clones (Fig. S4 G). Quantitatively, the top 10 T_Ly49_ clonotypes accounted for ∼30% of the total T_Ly49_ population, indicating moderate clonal expansion but not oligoclonal dominance (Fig. S4 G). While MHC class Ib–restricted CD8^+^ regulatory T cells (Tregs) exhibit a near-monoclonal TCR repertoire dominated by TRAV9N3 (Vα3.2) and TRBV12-1/12-2 (Vβ5.1/5.2) (Kim et al., 2024), ∼30% of tumor-specific T_Ly49_ cells in our MC38 tumors expressed TRAV9N3—a frequency higher than in other intratumoral subsets but far below that of MHC Ib–restricted Tregs (Fig. S4 H). These data suggest that tumor-specific T_Ly49_ cells likely recognize a broader and more diverse set of tumor antigens rather than being restricted to a narrow set of MHC class Ib–presented self-peptides.

Emerging evidence suggests that T_Ly49_ cells suppress T cell response and play a key role in maintaining self-tolerance (Li et al., 2022). To assess the suppressive function of tumor-specific T_Ly49_ cells, we cocultured tumor-specific T_Ly49_ with responder T_EFF_ in vitro. Compared with nonsuppressive T_EM_ controls, T_Ly49_ significantly increased cell death of T_EFF_ (Fig. S4, I and J) and suppressed proliferation of CTV-labeled responder T cells (Fig. S4, K–M). To examine its function in vivo, we isolated tumor-specific T_Ly49_ cells (Ly49E/F^+^ 2B4^+^ PD1^+^ P14 cells) from tumor tissues and adoptively transferred them into MC38-gp33 tumor-bearing mice. Intratumoral T_EM_/T_EFF_ cells were also isolated and transferred as a control. While T_EM_/T_EFF_ cells inhibited tumor growth, tumor-specific T_Ly49_ cells opposingly promoted tumor growth, indicating the regulatory function of T_Ly49_ cells in suppressing in vivo antitumor responses (Fig. S4 N). In addition, intratumor T_Ly49_ cells exhibited a gain of exhaustion signatures compared with T_EM_ and T_EFF_ subsets, implying they might be targeted by immune checkpoint blockade (Fig. S4 O).

**Figure S5.**
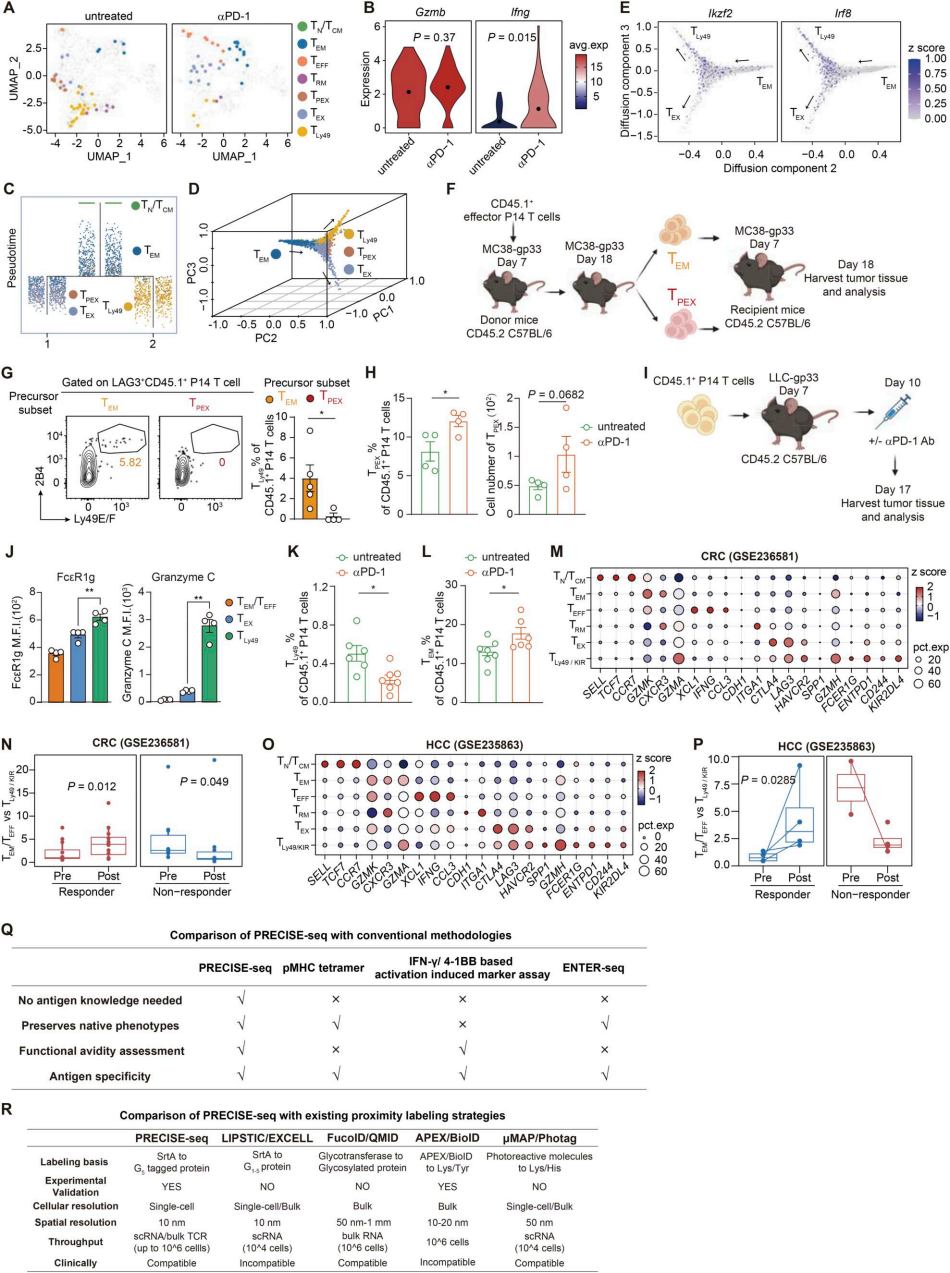
**Tumor-specific T cells are skewed toward a regulatory T**_**Ly49**_ **state, and the formation of which is attenuated by αPD-1 therapy. (A)** UMAP displaying the phenotypic distribution of high-confidence tumor-specific T cells in untreated tumors (left) and tumors treated with αPD-1 Ab (right). **(B)** Transcriptomic analysis of cytokine expression in tumor antigen-specific (Biotin^+^) CD8^+^ T cells derived from MC38 tumor-bearing mice with or without αPD-1 Ab treatment. **(C)** Inferred differentiation trajectory among five CD8^+^ T cell subsets from PRECISE-seq by URD analysis. **(D)** Inferred differentiation trajectory among CD8^+^ T cell subsets from PRECISE-seq by diffusion map algorithm. **(E)** Gene expression of *Ikzf2* and *Irf8* along the differentiation trajectory. **(F)** Schematic illustration for investigating the precursor of T_Ly49_ cells in vivo. Two progenitor T cells, namely, T_EM_/T_EFF_ (LAG3^−^) and T_PEX_ (LAG3^+^ 2B4^−^), were isolated from tumor tissues using FACS and subsequently intratumorally injected into MC38-gp33 tumor-bearing mice 7 days after tumor inoculations. The tumor-infiltrating CD45.1^+^ P14 T cells were harvested for subsequent phenotype profiling by flow cytometry on day 18. (**G**) Representative flow plots (left) and bar plots (right) illustrating the frequencies of T_Ly49_ within tumor-infiltrating CD45.1^+^ P14 T cell following adoptive transfer of T_EM_ or T_PEX_, respectively. Summary of two independent experiments and *n* = 4 independent animals. Data are presented as the mean ± SEM, and results were compared by unpaired, one-tailed Student’s *t* test. * represents P < 0.05. (**H**) Bar plots illustrating the frequencies and absolute number of T_PEX_ (PD-1^+^ Ly108^+^ Tim-3^−^) in tumor-infiltrating CD45.1^+^ P14 T cells with or without αPD-1 Ab treatment. **(I)** Experimental design for phenotypic profiling of tumor-specific T cells under αPD-1 Ab treatment within murine LLC tumor models. CD45.2 C57BL/6 mice were inoculated subcutaneously (s.c.) with LLC-gp33 tumor cells (5 × 10^5^) and received i.v. adoptive transfer of activated CD45.1^+^ P14 T cells (2 × 10^5^) 7 days after tumor inoculation. The LLC-gp33 tumor-bearing mice were randomly grouped based on the tumor size (*n* = 6 mice per group) and treated with 200 μg αPD-1 Ab 10 days after tumor inoculation. Tumor-infiltrated CD45.1^+^ P14 T cells were harvested and subjected to phenotypic profiling by flow cytometry on day 17. **(J)** Bar plots showing the expression of FcεR1g and granzyme C on T_Ly49_, T_EX_, and T_EM_/T_EFF_ within tumor-infiltrating CD45.1^+^ P14 T cells derived from the untreated group. **(K)** Bar plots depicting the reduction in frequencies of T_Ly49_ within tumor-infiltrating CD45.1^+^ P14 T cells under αPD-1 therapy. **(L)** Bar plots depicting an increase in the frequency of T_EM_ within tumor-infiltrating CD45.1^+^ P14 T cells under αPD-1 Ab. **(M)** z score of the average expression of marker genes across each CD8^+^ T cell subset in the CRC cohort (GSE236581). **(N)** Box plot showing the T_EM_/T_EFF_-to-T_Ly49/KIR_ cell ratio before and after αPD-1 treatment in responders and nonresponders in the CRC cohort (GSE236581). The P value was determined by a two-sided Wilcoxon rank-sum test. **(O)** z score of the average expression of marker genes across each CD8^+^ T cell subset in the HCC cohort. **(P)** Box plot showing the T_EM_/T_EFF_-to-T_Ly49/KIR_ cell ratio before and after PD-1 blockade treatment in responders and nonresponders in the HCC cohort. The P value was determined by a two-sided Wilcoxon rank-sum test. **(Q)** Table for comparison of PRECISE-seq with conventional methodologies. **(R)** Table for comparison of PRECISE-seq with existing proximity labeling strategies. Data are representative of *n* = 4–6 independent animals, unless otherwise noted. Each dot represents an individual biological replicate. Data are presented as the mean ± SEM, and results were compared by unpaired, two-tailed Student’s *t* test. * represents P < 0.05; ** represents P < 0.01. LLC, Lewis lung carcinoma.

We next examined the phenotypic dynamics of tumor-specific T cells in response to αPD-1 treatment. We observed an increase in the T_EX_ subset, potentially due to the conversion of T_PEX_ into a terminally exhausted phenotype upon αPD-1 treatment (Fig. 6 E and Fig. S5 A), consistent with the essential role of T_PEX_ in this process (Miller et al., 2019). We noticed that following αPD-1 Ab treatment, the frequency of tumor-specific T_Ly49_ was markedly reduced, and most tumor-specific T cells acquired effector-like phenotypes, segregating into T_EFF_ and T_EM_ compartments. Such enhanced formation of effector subsets was consistent with the increased production of effector cytokines in Biotin^+^ CD8^+^ T cells after αPD-1 Ab treatment (Fig. S5 B and Fig. S3 M). This indicates that PD-1 blockade can direct tumor-specific T cells into an effector state. Given the dramatic reduction of T_Ly49_ following PD-1 blockade, we next investigated the function and origin of this specific subset. The URD algorithm revealed strong directional flows originating from less-differentiated T_CM_/T_EM_ populations, which bifurcate into either T_EX_ or T_Ly49_ cells (Fig. S5 C). Specifically, the T_Ly49_ trajectory diverged early from T_EM_ and followed a differentiation pathway distinct from T_EX_ (Fig. S5, D and E). Further experimental validation supports the differentiation analysis, demonstrating that T_EM_ cells serve as progenitors of T_Ly49_ cells, independent of the T_EX_ differentiation trajectory (Fig. S5, F and G).

We next validated the effect of αPD-1 Ab on T cell fate commitment by transferring P14 cells into MC38-gp33 tumor-bearing mice followed by αPD-1 Ab treatment (Fig. 6 F). Consistent with the established notion that T_PEX_ expansion is a key mode of action for αPD-1 efficacy, αPD-1 Ab treatment indeed increased the absolute number of T_PEX_ cells within tumors (Fig. S5 H). Noticeably, αPD-1 Ab treatment led to a significant reduction in the frequency and absolute cell number of regulatory T_Ly49_ cells (Fig. 6, G and H). Conversely, we observed a concomitant rise in the frequency and absolute number of CXCR3^+^ PD1^−^ T_EM_ cells after αPD-1 treatment (Fig. 6, I and J), indicating a shift toward antitumor effector branch. Similar results were also observed in experiments with mice bearing LLC-gp33 tumors (Fig. S5, I–L). Together, these findings demonstrate that PD-1 blockade not only expands T_PEX_ cells but also disrupts the balance between regulatory T_Ly49_ cells and antitumor T_EM_ cells.

### The phenotypic shift from T_Ly49/KIR_ to T_EM_/T_EFF_ state correlated with responsiveness to immunotherapy

**Figure 7.**
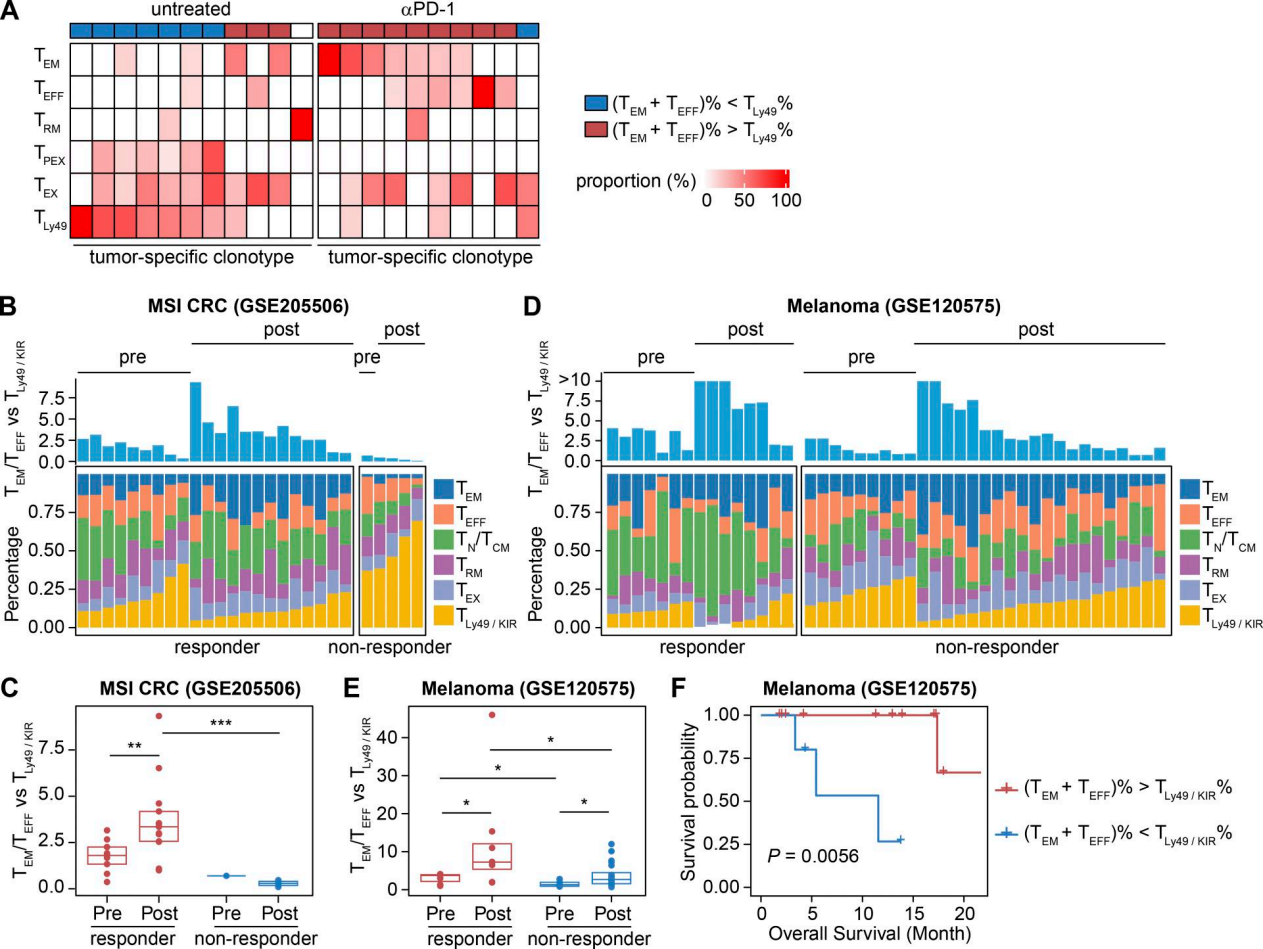
**Immunotherapy responsiveness prediction based on the phenotypic transition from the T**_**Ly49/KIR**_ **state to the T**_**EM**_ **/T**_**EFF**_ **state. (A)** Heatmap showing the phenotypic composition of each tumor-specific clonotype. **(B)** Phenotypic distribution of CD8^+^ T cells in each tumor sample from CRC patients receiving immunotherapy, predicted based on their similarity to T cell subsets identified in the MC38 tumor model. These patients were treated with toripalimab (αPD-1 Ab) with or without celecoxib before curative surgical resection. The T_EM_/T_EFF_-to-T_Ly49/KIR_ ratio is shown on the top. Samples before and after treatment are shown together. To enhance discrimination, the T_PEX_ subset in the MC38 model was merged into the T_EX_ subset to construct the reference profile for prediction. **(C)** Box plot showing the T_EM_/T_EFF_-to-T_Ly49/KIR_ ratio before and after PD-1 blockade treatment in responders and nonresponders in the MSI CRC cohort. One pretreatment sample, which had no predicted T_Ly49/KIR_ and exhibited an infinite ratio, was not included in the display. The P value was determined by a two-sided Wilcoxon rank-sum test. ** represents P < 0.01. *** represents P < 0.001. **(D)** Phenotypic distribution of CD8^+^ T cells in each tumor sample from melanoma patients receiving immunotherapy (bottom). These patients were treated with αPD-1 Ab with or without αCTLA-4 Ab. The T_EM_/T_EFF_-to-T_Ly49/KIR_ ratio is shown on the top. Samples before and after treatment are shown together. **(E)** Box plot showing the T_EM_/T_EFF_-to-T_KIR_ ratio before and after PD-1 blockade treatment in responders and nonresponders in the melanoma cohort. The P value was determined by a two-sided Wilcoxon rank-sum test. * represents P < 0.05. **(F)** Kaplan–Meier survival curve for the overall survival of 17 melanoma patients. Patients were divided into two groups based on the pretreatment T_EM_/T_EFF_-to-T_Ly49/KIR_ ratio (ratio >1: *n* = 12; ratio <1: *n* = 5). A P value was calculated with a two-tailed log-rank test.

We hypothesized that an elevated ratio of T_EM_/T_EFF_ to T_Ly49_ cells could indicate an effective antitumor response following immune intervention. To explore this further, we calculated the T_EM_/T_EFF_-to-T_Ly49_ cell ratio for individual clonotypes and categorized them into two groups: those displaying a higher T_EM_/T_EFF_ percentage than T_Ly49_, and those with a lower ratio. Treatment with αPD-1 Ab favored a T_EM_/T_EFF_ phenotype, with a T_EM_/T_EFF_-to-T_Ly49_ cell ratio >1 in 9 out of 10 tumor-specific clonotypes (Fig. 7 A). In summary, PRECISE-seq facilitated the monitoring of tumor-specific clonotypes within the TME, revealing a phenotypic shift from T_Ly49_ to T_EM_/T_EFF_ state in tumor-specific clonotypes following immune intervention.

To determine whether these insights about the phenotypic shift in response to PD-1 blockade can translate to the effectiveness of immunotherapies in human cancers, we collected publicly available scRNA-seq datasets from patients undergoing PD-1 blockade and assigned a cellular state for each CD8^+^ T cell based on their similarity to the CD8^+^ T cell subsets identified in PRECISE-seq data (Materials and methods). We analyzed four clinical cohorts across various cancers: (1) mismatch repair–deficient CRC (MSI CRC) (Li et al., 2023); (2) locally advanced or metastatic CRC (Chen et al., 2024); (3) patients with unresectable HBV^+^ HCC (Guo et al., 2025); and (4) metastatic melanoma (Sade-Feldman et al., 2018). In the MSI CRC cohort, immunotherapy resulted in a decrease in T_Ly49_-like CD8^+^ T cells (T_Ly49/KIR_) and an increase in T_EM_/T_EFF_ cells among responders (Fig. 7 B). The T_EM_/T_EFF_-to-T_Ly49/KIR_ ratio significantly rose in these samples after treatment (Fig. 7 C). Conversely, in nonresponders, the T_EM_/T_EFF_-to-T_Ly49/KIR_ ratio remained notably lower than in responders after treatment, suggesting that a preference for the T_Ly49/KIR_ state was associated with resistance to immunotherapy. Such an association with response to immune checkpoint blockade was also observed in an independent CRC cohort and an HCC cohort, further supporting that the phenotypic shift from T_Ly49/KIR_ to T_EM_/T_EFF_ state may play a critical role in mediating the therapeutic effects of the PD-1 blockade (Fig. S5, M–P).

Within the melanoma cohort, the T_EM_/T_EFF_-to-T_Ly49/KIR_ ratio increased following αPD-1 treatment for both responders and nonresponders (Fig. 7, D and E). However, this ratio was significantly lower in nonresponders compared with responders following treatment. Notably, even prior to treatment, responders exhibited a higher T_EM_/T_EFF_-to-T_Ly49/KIR_ ratio than nonresponders, indicating a preexisting effective antitumor response. We discovered that patients with a ratio >1 had longer survival than those with a ratio <1 (log-rank, P = 0.0056, Fig. 7 F), suggesting that the T_EM_/T_EFF_-to-T_Ly49/KIR_ ratio could serve as a predictive pretreatment marker for clinical benefits from αPD-1 treatment in melanoma patients. In summary, in line with previous observations, our findings revealed that effective antitumor responses necessitate a phenotypic shift from a regulatory T_Ly49/KIR_ state toward an effector state.

## Discussion

Recent advances in T cell–based immunotherapy have transformed cancer treatment, yet heterogeneous patient responses highlight the need to better define T cell states and behaviors following therapy. Here, we establish PRECISE-seq, which integrates SrtA-catalyzed proximity labeling with single-cell sequencing to jointly profile antigen specificity, TCR potency, and phenotypic states. PRECISE-seq sensitively detects antigen-specific T cells, including low-frequency CMV- and tumor-specific clonotypes, while preserving native cellular states. Using this approach, we identify a regulatory T_Ly49_ state enriched among tumor-specific T cells and demonstrate that PD-1 blockade promotes their transition toward an effector-like state associated with improved therapeutic responsiveness. These findings provide insight into how immune checkpoint blockade reshapes T cell fate in tumors.

Existing approaches largely rely on pMHC-TCR binding measurements or activation-based functional screens (Ma et al., 2021; Newell et al., 2013; Yu et al., 2022), which either fail to capture higher order cellular context (Sibener et al., 2018) or disrupt endogenous phenotypes (Oliveira et al., 2021; Yu et al., 2022) (Fig. S5Q). Although proximity labeling strategies have been developed, nonspecific or long-range labeling limits precision (Liu et al., 2022; Liu et al., 2020; Oslund et al., 2022; Zhang et al., 2023) (Fig. S5R). PRECISE-seq addresses these limitations through short-range SrtA-mediated ligation confined to the immunological synapse (Oakley et al., 2022) and uniform chemical conjugation of labeling substrates, enabling accurate capture of contact-dependent TCR recognition (Nakandakari-Higa et al., 2024; Zhang et al., 2022). Antigen specificity is inferred directly from T cell–APC contacts, without the need for predefined epitope information or engineered receptor systems, making it uniquely suited for translational studies. Importantly, labeling intensity provides a quantitative measure of relative functional avidity in situ, representing a distinguishing feature of PRECISE-seq. Moreover, recording occurs without in vitro activation or expansion, thereby preserving endogenous phenotypic states.

Our analysis further reveals that tumor-specific T cells preferentially adopt a T_Ly49_ program characterized by inhibitory lectin-like receptor expression, exhaustion features, and immunoregulatory activity distinct from previously described NK-like effector states (Beltra et al., 2023). These findings complement recent studies demonstrating exhaustion features in tumor-specific T cells within the TME (Lowery et al., 2022). Functionally, we demonstrate that these cells directly suppress antitumor immunity by inducing cell death and inhibiting proliferation of T_EFF_, providing a mechanistic basis for their protumoral effects in vivo. PD-1 blockade induces a transcriptional shift from T_Ly49_ toward an effector phenotype, consistent with improved clinical outcomes in immunotherapy cohorts. While our study provides a new mode of action for αPD-1 therapy, the mechanisms underlying the regulation of T_Ly49_ differentiation and their functional role in tumors could be explored in follow-up studies.

By enabling concurrent mapping of antigen specificity, interaction strength, and cell state, PRECISE-seq provides a framework for dissecting T cell responsiveness in vivo and informs the mechanistic understanding of immune interventions, supporting more precise immunotherapeutic strategies. In adoptive T cell therapy, PRECISE-seq may facilitate the identification of optimal TCR candidates, as quantitative assessment of interaction strength highlights clonotypes with superior potency and favorable phenotypes, such as effector- or memory-like states. In summary, PRECISE-seq represents a significant advancement in our understanding of T cell biology, immunotherapy response, and disease pathogenesis. It opens new avenues for developing innovative therapeutic interventions and personalized treatment strategies.

## Materials and methods

### Mice

C57BL/6 mice were purchased from Charles River Laboratories. C57BL/6 congenic CD45.1 mice and CD45.1 OT-I (OT-I) mice were kindly provided by Professor Xiaohuan Guo (Tsinghua University, Beijing, China). CD45.1 P14 mice were kindly provided by Professor Hai Qi (Tsinghua University, Beijing, China). Spp1-CreERT2-IRES-EGFP mice (strain no. T049832) were purchased from GemPharmatech. Rag2^−/−^ mice were purchased from the Jackson Laboratory. All mice were bred and housed under pathogen-free conditions at Tsinghua University, Beijing, China. Mice of 6–10 wk of age were used for all animal experiments. All mice were used in accordance with Tsinghua University Animal Ethics Committee guidelines.

### Peptide

Unless otherwise noted, all peptides used in this study were synthesized by SciLight Biotechnology, with a purity exceeding 95%. The chemical structure of Biotin-LPETG*G (G* refers to 2-hydroxyacetic acid) was described in the previous study (Ge et al., 2019; Williamson et al., 2012). The NHS-PEG4-maleimide was purchased from Confluore Biological Technology Co., Ltd. Sequences of all peptides are included in Table S1.

### Plasmid

For the plasmid constructs transfected into HEK293T cells, the CD40L and SrtA proteins were cloned into the PLVX vector encoding a fluorescent reporter (tdTomato) and a P2A linker at the N terminus. The SrtA protein with an N-terminal Flag tag was directed to anchor on the cell membrane through pDisplay system, and the protein sequence was described in the previous study (Pasqual et al., 2018). This tdTomato-P2A-pDisplay SrtA plasmid was also used in the lentivirus package to construct a SrtA-expressing stable cell line. A bicistronic gene and SrtA protein were inserted into the downstream of CD40L protein to co-express these two proteins on the cell surface. The CD40 protein was cloned into a PLVX vector with a C-terminal GFP reporter fusion. The 1G4-TCR cloned into the pHAGE-IRES-RFP vector was obtained from Professor Xin Lin’s group (Tsinghua University, Beijing, China). The CMV pp65_495–503_-specific TCR sequence (clone 4, clone 40, clone 52, and clone 67), synthesized by RootPath, Inc., was cloned into the pHAGE vector. The CD19 sequence was cloned between the TCRα chain and TCRβ chain via P2A self-cleaving peptide linker, allowing CD19 to serve as a co-expressed surrogate marker for monitoring TCR surface expression. The mouse tumor-reactive TCRs were cloned into a retroviral expression vector (RVKM), which encodes an EGFP and a P2A linker at the N terminus. For SrtA protein expression and purification in *Escherichia coli*, the SrtA was cloned into a pET-28a^+^ vector with a C-terminal 6 × His tag.

### Cell line and PBMC isolation

The K562-HLA-A2 cells and TCR-deficient Jurkat T cell line, a kind gift from Professor Xin Lin (Tsinghua University, Beijing, China), were cultured in RPMI 1640 complete medium (Thermo Fisher Scientific) containing 10% fetal bovine serum (FBS, Gemini), 25 mM HEPES, and 1% nonessential amino acids. B16-OVA is an OVA-transfected clone derived from the mouse melanoma cell line B16-F10 (ATCC, CRL-6475) as previously described (Han et al., 2019). LLC (CRL-1642; ATCC) is a Lewis lung carcinoma cell line. A MC38 cell line was derived from chemically induced grade III adenocarcinoma in C57BL/6 mice (Deng et al., 2014), and B2M-deficient MC38 tumor cells were obtained from Dr. Deng Pan (Tsinghua University, Beijing, China). All cell lines were cultured in an incubator at 37°C under 5% CO_2_ and verified to be free of *Mycoplasma* contamination. PBMCs collected from HLA-A2^+^ CMV-seropositive healthy donors were obtained from Peking University Cancer Hospital with consent forms. PBMCs were isolated by density gradient centrifugation using Ficoll (Thermo Fisher Scientific) and cryopreserved in a freezing medium containing 90% FBS and 10% DMSO. HLA typing was performed by staining with anti-HLA-A2-APC (clone BB7.2; BioLegend).

### Lentiviral transduction

Lentiviral transduction was performed as described previously (Liu et al., 2021). In brief, HEK293T cells were plated on 6-cm dishes at a density of 1 × 10^6^ cells per plate 24 h before transfection. HEK293T cells were cotransfected with 3 μg of the lentiviral plasmid, 1.5 μg of envelope vector (pMD2.G), and 1 μg of packaging vector (psPAX2). The culture medium was replaced 8 h after transfection, and the viral supernatant was collected 48 h or 72 h later. The viral supernatant was filtered through a 0.45-μm PES Syringe Filter (Thermo Fisher Scientific) and then used to infect tumor cells in the presence of 10 μg/ml polybrene (Sigma-Aldrich). Tumor cells, including B16-OVA, MC38, and K562-HLA-A2, were infected with lentivirus to overexpress SrtA protein on the cell surface. The SrtA expression was assessed by staining with an anti-Flag tag (anti-DYKDDDDK) Ab (clone L5; BioLegend), and the tumor clones with high levels of SrtA expression were sorted. Both MC38-zsGreen-LCMVgp_33–41_ (MC38-gp33) and LLC-zsGreen-LCMVgp_33–41_ (LLC-gp33) are single-cloned cell lines, generated by sorting zsGreen-positive cells after lentiviral transduction with a construct expressing zsGreen fused to six tandem repeats of the LCMVgp_33–41_ epitope (KAVYNFATM). A TCRαβ-deficient Jurkat T cell line was transduced with the viral supernatant expressing 1G4-TCR, and TCR expression was assessed based on the co-expression of CD3 and RFP. To generate CMV-specific TCR-expressing cells, TCRαβ-deficient Jurkat cells were transduced with a bicistronic lentiviral vector encoding a CMV pp65-specific TCR coupled to a CD19-P2A selection marker. TCR-positive populations were subsequently isolated by fluorescence-activated cell sorting (FACS) based on the dual surface expression of CD3 and CD19.

### AP-HA tagging on cell surface

1 × 10^6^ live cells were suspended in 100 μl freshly prepared NHS-PEG4-maleimide solution (50 μM in sterile PBS) and incubated for 5 min at 37°C. Then, the labeling reaction was quenched with an equivalent volume of FBS-free 1640 medium, and the supernatant was removed after centrifugation. Subsequently, the cells were resuspended in PBS containing 200 μM AP-HA probe and incubated for 5 min at 37°C. Afterward, the cells were washed with complete 1640 medium. The AP-HA tag^+^ cells were then ready for subsequent labeling experiments or analysis. To confirm chemical reaction labeling efficiency, the AP-HA tag abundance was assessed by flow cytometry through staining with an anti-HA tag Ab (clone 16B12; BioLegend). The consistent 2-log increase in AP-HA tag ΔMFI served as a standardized internal quality control for substrate availability and a benchmark for cross-batch comparisons.

### Recognition reactivity assessment of the AP-HA tag by cell surface sortagging

The surface of HEK293T cells was conjugated with a homogeneous level of the AP-HA tag, and then, we performed the cell surface sortagging using the purified SrtA protein. The SrtA protein was expressed and purified from *E. coli* BL21 (DE3) cells. Sortagging reactions were performed in a total 100 μl volume at 37°C for 1 h in DMEM complete medium. 5 × 10^5^ AP-HA tag^+^ HEK293T cells were incubated with 20 μM purified SrtA protein and 100 μM Biotin-LPETGG probe. HEK293T cells after sortagging were washed three times and stained with streptavidin Ab (BioLegend), followed by flow cytometry analysis.

### Characterization of the impact of the AP-HA tag on T cell antigen sensitivity

3 × 10^5^ MC38-SrtA tumor cells were seeded into a 96-well U-bottom plate and pulsed with serially titrated OVA_257–264_ peptide ranging from 10^−5^ μM to 10 μM in RPMI 1640 complete medium for 2 h at 37°C. Following incubation, the supernatant was replaced with RPMI 1640 complete medium. Splenocyte suspensions were prepared by mechanical disruption of spleens from CD45.1 OT-I mice, followed by red blood cell lysis using RBC lysis solution (eBioscience). Half of the splenocytes were conjugated with the AP-HA tag, while the remaining splenocytes were left untreated. 1 × 10^6^ AP-HA–tagged or untreated splenocytes were added and cocultured with MC38-SrtA tumor cells pulsed with OVA_257–264_. As a negative control, splenocytes were also co-incubated with MC38-SrtA cells in the absence of antigen stimulation. Following a 2-h incubation at 37°C, the expression of CD69 (clone FN50; BioLegend) on the CD45.1^+^ OT-I T cell was assessed by flow cytometry.

### In vitro coculture labeling assay

SrtA^+^ aAPCs (3 × 10^6^ cells/ml) were pulsed with 1 mg/ml NY-ESO-1_157–165_ or CMV pp65_495–503_ in RPMI 1640 complete medium for 2 h at 37°C. After incubation, the SrtA^+^ aAPCs were washed once to remove redundant peptides. 1G4-JC5 cells were labeled with the AP-HA tag and resuspended with RPMI 1640 complete medium. Then, 1 × 10^5^ per well AP-HA tag^+^ 1G4-JC5 cells were seeded into 96-well U-bottom plates and incubated with SrtA^+^ aAPC preloading antigen at a ratio of 1:3 for 2 h at 37°C. The Biotin-LPETGG probe was added at the beginning of the coculture with a final concentration of 100 μM. After 2 h of incubation, the reaction was quenched with FACS buffer (PBS containing 2% FBS and 1 mM EDTA), and then, cells were harvested and washed twice with FACS buffer. Subsequently, cells were stained with Abs against the HA tag, Biotin, and CD69 (clone FN50; BioLegend), followed by flow cytometry analysis.

For the coculture assay of SrtA^+^ tumor cells and OT-I T cells, splenocyte suspensions were obtained by the mechanical disruption of spleens from OT-I mice, and red blood cells were then lysed in RBC lysis solution (eBioscience). Following AP-HA tag labeling, 1 × 10^6^ splenocytes were incubated with 3 × 10^5^ B16-OVA-SrtA or B16F10-SrtA in the presence of the 100 μM Biotin-LPETGG probe in a 96-well U-bottom plate. After 2 h of incubation at 37°C, cell mixtures were then stained and analyzed by flow cytometry. For the APL assay, MC38-SrtA tumor cells (3 × 10^6^ cells/ml) were pulsed with 10 μM APL in RPMI 1640 complete medium for 2 h at 37°C. After APL peptide loading, the MC38-SrtA tumor cells were washed once to remove redundant peptides for subsequent co-incubation experiments. To evaluate the sensitivity of PRECISE labeling, CD45.1^+^ OT-I T cells were serially diluted with CD45.2^+^ polyclonal CD8^+^ T cells at ratios ranging from 1:10 to 1:10,000. The T cell mixtures were conjugated with the AP-HA tag and subsequently cocultured with B16-OVA-SrtA tumor cells in the presence of a 100 μM Biotin-LPETGG probe in a 96-well U-bottom plate. Following a 2-h incubation at 37°C, the cell mixtures were then stained and analyzed by flow cytometry.

### Characterization for the variability of AP-HA tag labeling in PRECISE-seq

1 × 10^6^ splenocytes derived from CD45.1 OT-I mice were incubated with freshly prepared NHS-PEG4-Mal solutions at concentrations of 0, 10, 25, and 50 μM in a total volume of 100 μl for 5 min at 37°C. After the removal of the supernatant, the cells were resuspended in PBS containing 200 μM AP-HA probe and incubated for an additional 5 min at 37°C. Subsequently, the cells were washed with RPMI 1640 complete medium, and the abundance of the AP-HA tag was assessed by flow cytometry using an anti-HA tag Ab (clone 16B12; BioLegend). To investigate the impact of AP-HA tag abundance on labeling efficiency, 1 × 10^6^ splenocytes expressing distinct levels of AP-HA tag abundance were incubated with 3 × 10^5^ B16-OVA-SrtA in the presence of 100 μM Biotin-LPETGG probe in a 96-well U-bottom plate. After a 2-h incubation at 37°C, Biotin labeling signals on CD45.1^+^ OT-I T cells were analyzed by flow cytometry.

### Biotin labeling of CMV-specific CD8^+^ T cells by PRECISE-seq

SrtA^+^ aAPCs (3 × 10^6^ cells/ml) were pulsed with 1 mg/ml CMV pp65_495–503_ peptide in RPMI 1640 complete medium for 2 h at 37°C. After incubation, the SrtA^+^ aAPCs were washed once to remove redundant peptides and resuspended in RPMI 1640 complete medium. Cryopreserved PBMCs were thawed and conjugated with the AP-HA tag as previously described. Following AP-HA conjugation, a consistent 2-log increase in AP-HA tag ΔMFI was measured by flow cytometry. This served as a standardized internal quality control for substrate availability and a benchmark for cross-batch comparisons. 1 × 10^6^ per well AP-HA tag^+^ PBMCs were seeded into 96-well U-bottom plates and incubated with 3 × 10^5^ SrtA^+^ aAPCs loading with CMV pp65_495–503_ peptide in the presence of 100 μM Biotin-LPETGG probe at 37°C. After 2 h of incubation, cells were washed with FACS buffer and stained with Abs against CD8 (clone RPA-T8; BioLegend), HA tag, Flag tag, and Biotin. For single-cell transcriptomic analysis, stained cells were further incubated with DNA-barcoded sample hashtag Abs (clone LNH-94; 2M2; BioLegend) for 20 min in PBS. Prior to the cell sorting procedure, 7-aminoactinomycin D (7-AAD) (BD Bioscience) was added to exclude dead cells. The Biotin^+^ CD8^+^ T cells, as well as total CD8^+^ T cells without AP-HA tag labeling, were sorted for PRECISE-seq and bulk TCR-seq analysis. For sorting of total CD8^+^ T cells, live CD8^+^ single cells were gated. For sorting of Biotin^+^ CD8^+^ T cells, live CD8^+^HA tag^+^ Biotin^+^ single cells were gated. A stringent gating threshold was established to maximize the detection of true positives while minimizing background noise. CD8^+^ T cells with Biotin intensity exceeding 10^3^ were defined as the Biotin^+^ population and sorted for sequencing.

### Assessment of TCR repertoire alteration in PRECISE-seq by bulk TCR-seq

SrtA^+^ aAPCs were pulsed with 1 mg/ml CMV pp65_495–503_ peptide in RPMI 1640 complete medium at a density of 3 × 10^6^ cells/ml and incubated for 2 h at 37°C. Following incubation, the SrtA^+^ aAPCs were washed once to remove excess peptides and resuspended in RPMI 1640 complete medium. PBMCs were previously isolated from HLA-A2^+^ healthy donors with CMV infection and identified by staining with HLA-A*02:01-CMV NLV-tetramer-PE (HG08T15006; Helixgen). Cryopreserved PBMCs were thawed, and half of the cells were conjugated with the AP-HA tag as previously described, while the remaining PBMCs were left untreated to serve as a baseline control. AP-HA–tagged PBMCs (1 × 10^6^ cells per well) were seeded into 96-well U-bottom plates and incubated with 3 × 10^5^ SrtA^+^ aAPCs loaded with CMV pp65_495–503_ at 37°C with 5% CO_2_ for 2 h. Both cultured AP-HA^+^ PBMCs and untreated PBMCs were collected and stained with anti-CD8-BV421 (301036; BioLegend). Before sorting, 7-AAD was added to discriminate between live and dead cells. Live CD8^+^ T cells were sorted and subsequently processed for bulk TCR-seq.

### Validation of CMV-specific TCR by the TCR-pMHC activation screening assay

Antigen specificity screening of CMV-specific TCRs was conducted by RootPath, Inc., with the experimental process performed as previously described (Moravec et al., 2025). Paired full-length TCRα and TCRβ chains, separated by a P2A linker, were synthesized and cloned into AAV vectors (PackGene). Healthy donor T cells were isolated using CD3 isolation kits (STEMCELL Technologies) and activated with CD3/CD28 beads (Thermo Fisher Scientific) in ImmunoCult Human T Cell Expansion media (STEMCELL Technologies), supplemented with 200 U/ml IL-2 (PeproTech). 2 days after bead activation, T cells were electroporated (program EO-115) with Cas9 ribonucleoprotein (RNP) using P3 buffer and Nucleofector 4D Electroporation System (Lonza). RNPs consisted of SpyFi Cas9 nuclease (Aldevron) duplexed with HPLC-purified single guide RNAs (Integrated DNA Technologies) targeting the human TRAC and TRBC1/2 loci. After electroporation, TCR knockout T cells were transduced with TCR library AAV (5 × 10^4^ genome copies per cell) and cultured for an additional 2 days. Beads were then removed, and cells were maintained in culture for 6–10 days.

For TCR discovery screens, TCR library-engineered T cells were cocultured overnight in duplicate with K562-HLA-A2 cells pulsed with CMV pp65_495–503_ peptide at an effector-to-target (E:T) ratio of 1:1. After coculture, reactive T cells were sorted based on the expression of T cell activation marker 4-1BB (clone 4B4-1; BioLegend). Sorted T cells were subjected to bulk TCR-seq. The quality control and alignment of sequencing reads have been described previously (Moravec et al., 2025). CMV-specific clonotypes were defined as those significantly enriched (FDR <0.05 and OR >4) in the CMV pp65_495–503_-pulsed group, compared with both the mock-pulsed and baseline groups, as determined by Fisher’s exact test.

In the IFN-γ ELISpot assay, paired full-length TCRα and TCRβ chains, separated by a P2A linker, were synthesized and electroporated in TCR-deficient T cells in PBMCs. 24 h after TCR mRNA electroporation, transient TCR-T cells were costained with anti-human TCRα/β receptor Ab (IP26) and anti-human CD137 (4B4-1) Ab, followed by flow cytometry analysis for TCR and CD137 cell surface expression as quality control of electroporation.

In coculture for the IFN-γ ELISpot assay, HLA-A2 mRNA was electroporated into K562 cells. After 3 h, cells were loaded with the tested peptide for 2 h. HLA-expressing K562 cells without peptide pulsing were used as HLA-only negative control. Individual TCRs were introduced into donor T cells via mRNA electroporation as described above. 6 h after electroporation, TCR-expressing T cells were cocultured individually with different APCs for 18 h, followed by IFN-γ ELISpot analysis. TCR-T only was added as a negative control. T cells expressing TCRs PMEL or C4DLT were cocultured with HLA-A2–expressing K562 cells pulsed with peptide gp100 and WT1.

### TCR avidity validation by the in vitro peptide stimulation assay

CMV-specific TCR-expressing cells were seeded at a density of 1 × 10^5^ cells per well in a 96-well U-bottom plate and cocultured with K562-HLA-A2 cells at an E:T ratio of 1:1. The CMV pp65_495–503_ peptide was serially diluted in RPMI 1640 complete medium and added to the cell mixture to achieve final concentrations ranging from 10^−7^ μM to 100 μM. Following a 2-h incubation at 37°C with 5% CO_2_, the cells were collected and stained with anti-CD69 (clone FN50; BioLegend), anti-CD19 (clone HIB19; BioLegend), and anti-CD3 (clone UCHT1; BioLegend) for subsequent flow cytometry analysis.

### Biotin labeling of tumor antigen-specific T cells by PRECISE-seq

For single-cell suspension preparation of tumor-infiltrating immune cells, collected MC38 tumor tissues were enzymatically digested with 0.26 U/ml Liberase TL, 1 mg/ml collagenase D, and 0.1 mg/ml DNase I (Roche) at 37°C for 30 min. Cells were filtered through a 70-μm cell strainer and washed twice with FACS buffer. Both B16-OVA tumor tissues and draining lymph nodes were mechanically disrupted into single-cell suspension. Subsequently, single-cell suspensions were conjugated with the AP-HA tag as previously described and cocultured with cognate SrtA^+^ tumor cells in 96-well U-bottom plates at a ratio of 3:1 (3 × 10^5^ SrtA^+^ tumor cells per well). The Biotin-LPETGG probe was added at a final concentration of 100 μM. After 2 h of incubation, cells were washed twice with FACS buffer and stained with Abs against CD45 (clone 30-F11; BioLegend), CD8 (clone 53-6.7; BioLegend), CD44 (clone IM7; BioLegend), HA tag, Flag tag, and Biotin.

For single-cell transcriptomic analysis, stained cells were further incubated with DNA-barcoded sample hashtag Abs (clone M1/42; 30-F11; BioLegend) for 20 min in PBS, followed by 7-AAD staining. The Biotin^+^ CD8^+^ T cell populations and live non–AP-HA-tagged tumor-infiltrating CD44^+^ CD8^+^ T cells were sorted for PRECISE-seq. For sorting of Biotin^+^ CD8^+^ T cells, live CD45^+^CD8^+^CD44^+^HA tag^+^Biotin^+^ single cells were gated. The Biotin^+^ population was defined according to that on CD8^+^ T cells under incubation with B2M-deficient MC38-SrtA tumor cells.

### Flow cytometry and cell sorting

Flow cytometry was performed following the previously published paper with some modifications (Dong et al., 2021). For surface marker staining, the single-cell suspension was incubated with Fc Block Ab (clone 2.4G2; BioXCell) for 10 min, followed by staining with Zombie fixable viability dye (BioLegend) to exclude the dead cells. After a washing step, cells were stained for cell surface markers at 4°C for 20 min in PBS. Subsequently, cells were washed twice with PBS to remove dissociative fluorescent Abs for further flow cytometry analysis. For intracellular staining, cells were fixed with fixation buffer (BioLegend) on ice for 15 min and then permeabilized twice with diluted 1× Intracellular Staining Permeabilization Wash Buffer (10×) (BioLegend). Abs against IFN-γ (clone XMG1.2; BioLegend), granzyme B (clone QA16A02; BioLegend), FcεR1g (FCABS400F; Sigma-Aldrich), and granzyme C (clone SFC1D8; BioLegend) were added and incubated for 1 h on ice. Then, the cells were washed with permeabilization buffer before flow cytometry analysis. Flow cytometry data were collected on LSR II (BD) or Symphony and/or sorted on FACSAria II or FACSAria III cell sorters (BD Biosciences). Data were analyzed by FlowJo (Tree Star).

### Gating strategies for T_Ly49_ subset

The gating procedure began with the selection of single, live CD45^+^ leukocytes, followed by the identification of CD8^+^ T cells. Subsequently, within the Lag3^+^PD-1^+^Ly108^−^CXCR3^−^ CD8^+^ T cell subset, further subdivision was performed based on the expression of 2B4 and Ly49E/F. T_Ly49_ cells were defined as 2B4^+^ Ly49E/F^+^.

### Tumor growth and treatment

6- to 8-wk-old female C57BL/6 (CD45.2) mice were subcutaneously implanted with 5 × 10^5^ B16-OVA tumor cells. For adoptive T cell transfer, CD8^+^ T cells were isolated from the lymph nodes and spleens of CD45.1 OT-I mice using EasySep Mouse CD8^+^ T Cell Isolation Kit (STEMCELL). 5 × 10^5^ purified CD45.1^+^ OT-I T cells were intravenously injected into tumor-bearing CD45.2 mice 7 days after tumor inoculation.

3 × 10^5^ MC38 tumor cells were subcutaneously inoculated into C57BL/6 female mice. The length (a) and width (b) of the tumors were measured starting on day 6 and then every 2 days thereafter, and tumor volume was calculated with the formula (ab^2^)/2. When the average tumor size reached about 150–200 mm^3^, mice were randomly divided into two groups (*n* = 5–7 per group), and 200 μg αPD-1 Ab (BioXCell) was intraperitoneally injected at day 7 after tumor inoculation.

5 × 10^5^ MC38-gp33 or LLC-gp33 tumor cells were subcutaneously inoculated on CD45.2 C57BL/6 mice. On day 7 after inoculation, tumor-bearing mice received 4.5 Gy total body irradiation, followed by intravenous adoptive transfer of 2 × 10^5^ activated CD45.1^+^ P14 T cells. The tumor-bearing mice were randomly divided into two groups based on tumor volume (*n* = 5–7 per group), and 200 μg αPD-1 Ab (BioXCell) was intraperitoneally injected at day 10 after tumor inoculation.

### In vivo TIL-mediated tumor killing assay

Biotin^+^ and Biotin^−^ tumor-infiltrating CD8^+^ T cells from MC38 tumor-bearing CD45.1 mice were sorted as previously described. 1000 Biotin^+^ or Biotin^−^ CD8^+^ T cells suspended in PBS were intravenously injected into CD45.2 Rag2^−/−^ mice, followed by subcutaneous inoculation of MC38 tumor cells (3 × 10^5^ per mice). Tumor curves were monitored every 2 days from day 4 after tumor inoculation.

### Isolation and activation of CD45.1^+^ P14 T cells

CD45.1^+^ P14 cells were harvested from the spleen and lymph nodes of CD45.1 P14 mice and subsequently stimulated with 1 μg/ml LCMVgp33 peptide (KAVYNFATM) at a density of 1 × 10^8^ cells/ml in RPMI 1640 complete medium at 37°C for 1 h. Following the removal of excess LCMVgp33 peptide, the splenocytes were resuspended at a density of 5 × 10^6^/ml in RPMI 1640 complete medium supplemented with 100 IU/ml recombinant human IL-2 (BioLegend), 10% FBS, 25 mM HEPES, 1% nonessential amino acids, 0.1% 2-mercaptoethanol, 100 U/ml penicillin, and 100 μg/ml streptomycin. After 24 h of activation, P14 T cells were collected for retroviral transduction or adoptive transfer.

### Retrovirus production

HEK293T cells were seeded in 10-cm dishes at a density of 3 × 10^6^ cells per plate 24 h before transfection. HEK293T cells were cotransfected with 10 μg of retroviral plasmids (rvkm-TCRβ-P2A-TCRα-P2A-EGFP or rvkm-EGFP), 10 μg of pCL-Eco plasmid, and 40 μl of PEI (40,000 MW). The culture medium was replaced 8 h after transfection, and the viral supernatants were harvested at 48 or 72 h thereafter. The viral supernatants were filtered through a 0.45-μm PES Syringe Filter (Thermo Fisher Scientific) and either used immediately or cryopreserved at −80°C.

### Retroviral transduction

Retrovirus production was performed as described previously (Kurachi et al., 2017). Activated P14 T cells were seeded into a 24-well plate at a density of 1 × 10^6^ per well in RPMI 1640 complete medium supplemented with 4 μg/ml polybrene and 100 IU/ml IL-2. 1 ml of retroviral supernatants was added to the P14 T cell suspension, followed by gentle mixing through pipetting. The P14 T cells were then subjected to centrifugal transduction with the retrovirus at 2,000 *g* for 2 h. After centrifugation, the P14 T cells were incubated for an additional 4 h under standard culture conditions (37°C, 5% CO_2_). Subsequently, the viral supernatants was replaced with RPMI 1640 complete medium containing 100 IU/ml IL-2, and transfected P14 T cells were incubated for an additional 2 days. The surface expression of the transgenic TCR was evaluated by flow cytometry based on the expression of EGFP, and the EGFP^+^CD45.1^+^CD8^+^ T cells were then isolated using a cell sorter.

### In vivo and in vitro assays for antigen specificity validation of tumor-reactive T cells

C57BL/6 mice were subcutaneously inoculated with 3 × 10^5^ MC38 and received 4.5 Gy total body irradiation 7 days after inoculation. Within 12 h after irradiation, 5 × 10^5^ fluorescence-activated cell–sorted EGFP^+^CD45.1^+^CD8^+^ T cells (purity >95%) either antigen-specific TCRs or mock-GFP were intravenously transferred into MC38 tumor-bearing mice (*n* = 4–8 per group). The tumor tissue and draining lymph nodes were harvested 14 days after tumor inoculations, and the absolute numbers of transferred EGFP^+^CD45.1^+^CD8^+^ T cells were quantified by counting beads (BioLegend).

To validate the antigen specificity of tumor-reactive TCR in vitro, EGFP^+^CD45.1^+^CD8^+^ T cells expressing TCRs or mock-GFP were harvested from the tuDLN of MC38 tumor-bearing mice. These T cells were seeded at a density of 5 × 10^5^ cells per well in 96-well U-bottom plates and cocultured with 3 × 10^5^ MC38 tumor cells for 18 h. The expression of activation markers on EGFP^+^CD45.1^+^CD8^+^ T cells was evaluated by flow cytometry staining with Abs against CD69 (clone H1.2F3; BioLegend), CD25 (clone PC61; BioLegend), and IFN-γ (clone XMG1.2; BioLegend).

### Adoptive T cell transfer assay

For donor mouse preparation, CD45.2 C57BL/6 mice were subcutaneously inoculated with 5 × 10^5^ MC38-gp33 tumor cells. 7 days after inoculation, the tumor-bearing mice received 4.5 Gy total body irradiation, followed by intravenous adoptive transfer of 2 × 10^5^ activated CD45.1^+^ P14 T cells 7 days after inoculation. Tumor tissues were harvested 18 days after inoculation and enzymatically dissociated into single-cell suspensions.

For evaluation of T_Ly49_ in promoting tumor growth, T_Ly49_ (LAG3^+^2B4^+^Ly49E/F^+^) and T_EM_-T_EFF_ (LAG3^−^) populations were isolated from the tumor-infiltrated CD45.1^+^ P14 T cells by FACS using stringent gating criteria. Each subset (6,000 cells) was intratumorally injected into recipient mice bearing MC38-gp33 tumor. The recipient mice that received PBS injection without T cell transfer served as negative controls. Recipient mice were prepared by subcutaneous inoculation with 5 × 10^5^ MC38-gp33 tumor cells 7 days before injection and subsequent treatment with 4.5 Gy total body irradiation. Recipient mice were randomly assigned to three groups (*n* = 3–6 per group), and tumor growth was monitored every 2 days starting from day 7 after tumor inoculation.

To investigate the precursor of T_Ly49_, T_EM_ (LAG3^−^CXCR3^+^) and T_PEX_ (PD-1^+^Ly108^+^Tim-3^−^) were isolated from the tumor-infiltrating CD45.1^+^ P14 T cells by FACS. Each subset (6,000 cells) was intratumorally injected into recipient mice bearing MC38-gp33 tumor (*n* = 3 per group). The preparation of recipient mice has been previously described. Tumor growth was monitored every 2 days starting from day 7 after tumor inoculation. The phenotypic compositions of tumor-infiltrating CD45.1^+^ P14 T cells in recipient mice were assessed by flow cytometry 11 days after T cell transfer.

### scRNA-seq

Cell viability was examined before single-cell library preparation to ensure the high quality of the scRNA-seq and TCR profiling data. Over 90% of cells remained viable throughout the labeling and sorting processes, which minimized the risk of RNA degradation and ensured the generation of high-fidelity sequencing data. The libraries were prepared using Chromium Next GEM Single Cell 5′ GEM, Library & Gel Bead Kit version 1.1 (Cat: 1000165) purchased from 10X Genomics according to the protocols provided by the manufacturer. The target cell recovery for each library was aimed at 8,000 cells, and the libraries were sequenced on an Illumina HiSeq X Ten platform.

### Bulk TCR-seq

Libraries for TCR-seq were prepared from 10 ng of total RNA using SMARTer Human TCRα/β Profiling Kit version 2 (Takara Bio). 18 cycles were used for each of the two semi-nested PCR amplification steps. Sequencing was performed on an Illumina NovaSeq by multiplexed paired-read run with 2 × 251 cycles.

### Processing of PRECISE-seq data of human CD8^+^ T cells

Single-cell libraries contained samples from the same patient used in another study; Cell Hashing was employed to label cells from distinct samples (Stoeckius et al., 2018). The paired scRNA-seq and scTCR-seq data were processed with the “cellranger multi” pipeline by Cell Ranger 6.0. Briefly, scRNA-seq reads were aligned to the GRCh38 genome, while scTCR-seq reads were aligned to the human GRCh38 V(D)J reference (version 5.0.0). The intronic reads were also counted, as suggested by Cell Ranger. The “cellranger aggr” command was applied to aggregate TCR clonotype outputs from cellranger multi. The CITE-seq reads were counted using CITE-seq-Count version 1.4.5 (https://github.com/Hoohm/CITE-seq-Count). Downstream analysis was conducted using the Seurat R package, version 3.2.3 (Stuart et al., 2019). Genes detected in fewer than 3 cells were removed, while cells identified by both scRNA-seq and CITE-seq were retained. The CITE-seq data underwent normalization using the centered log-ratio transformation. The HTODemux function was employed to demultiplex samples based on the signals of hashtag oligos, which label sample origins. For the Biotin^+^ sample, predicted doublets were removed, and negative/ambiguous cells were subjected to a second round of demultiplexing to exclude definitively hashtag-negative cells. For the baseline sample, predicted singlets were retained.

### Quality control of PRECISE-seq data

After defining singlets, quality control was conducted to remove low-quality cells from the total cell population, including doublets and hashtag-negative cells. Cells were excluded if they expressed fewer than 300 genes or if the proportion of mitochondrial gene expression exceeded 15%. Subsequently, UMI counts were normalized using the NormalizeData function in Seurat, after excluding mitochondrial and ribosomal protein genes. Highly variable genes (HVGs) were defined as the top 2,500 variable genes, excluding those listed in the blacklist provided by a recent pan-cancer T cell study (Zheng et al., 2021). The effects of total UMI counts and the percentage of mitochondrial genes were regressed out. These HVGs were then utilized for principal component (PC) analysis. The optimal number of PCs was determined using the ElbowPlot function. Subsequently, the shared nearest neighbor graph was constructed with the FindNeighbors function with the selected number of top PCs, employing nn.method = “annoy” and annoy.metric = “cosine.” Subsequently, the Louvain algorithm was applied to cluster cells with a resolution setting of 0.3. Clusters expressing markers of non-T cells, such as myeloid, B, and erythroid cells, were removed. scTCR-seq data were then integrated to identify cells with detected TCRs, and clusters with a low proportion of cells with detected TCRs were subsequently excluded. For TCR profiling, clonotypes with frameshift mutations, stop codons, or > 2 TCRα or TCRβ chains (indicative of doublets) were excluded from downstream analysis. This ensured only functional and biologically relevant TCR sequences were retained. Mucosal-associated invariant T cells, known to recognize metabolites presented on MR1 and potentially skewing the analysis, were removed (Kjer-Nielsen et al., 2012).

### Integration and annotation of PRECISE-seq data of human CD8^+^ T cells

The scRNA-seq data were integrated with a single-cell atlas of PBMCs to define conserved human CD8^+^ T cell subsets (Mogilenko et al., 2021). The public scRNA-seq dataset of CD8^+^ T cells was downloaded from the Synapse repository (https://www.synapse.org; syn22255433). The log-scaled normalized data were converted to a count matrix and then processed through the preprocessing pipeline described above, including data normalization, scaling, and regression. Next, the public dataset was integrated with CD8^+^ T cells with detected TCR from PRECISE-seq, using the integration procedure in the Seurat R package. The public dataset and baseline CD8^+^ T cells were set as references. The Seurat pipeline, as described above, was executed to identify clusters. The CD8^+^ T cell clusters were then annotated based on the known label of CD8^+^ T cells from the public dataset. Subsequently, the expression levels of gene signatures were calculated using the AddModuleScore function in the Seurat package. The curated gene signatures applied to T cells were obtained from a recent pan-cancer T cell study (Chu et al., 2023).

### Annotate the antigen specificity and TCR potency of CMV-specific clonotypes

The TCR clonotype was defined as a unique combination of the CDR3α and CDR3β nucleotide sequences. Cells with detected paired TCRα and TCRβ were retained. To identify high-confidence CMV-specific clonotypes, we established a statistical enrichment strategy for the analysis of PRECISE-seq data. Clonotypes were considered to be enriched if they met these criteria: (1) a minimum cell number threshold of 2 in the Biotin^+^ group; (2) significant enrichment in the Biotin^+^ group compared with the baseline group, with an FDR of <0.05 by Fisher’s exact test; and (3) an OR >4.

### The definition and calculation of the potency score

The potency score of each TCR is defined as the relative likelihood of a TCR being enriched in the Biotin-labeled population compared with irrelevant TCRs. Irrelevant TCRs are defined as clonotypes with decreased frequencies in the Biotin^+^ group compared with the baseline group. Based on this definition, the relationship between the frequency of a TCR clonotype *i* in the Biotin^+^ group and the baseline group can be expressed as follows:

$$\frac{p_{i}^{Biotin}}{\sum p_{irrelevant}^{Biotin}}=S_{i}\;\cdot \;\frac{p_{i}^{baseline}}{\sum p_{irrelevant}^{baseline}}.$$

Here, $p_{i}^{Biotin}$ and $p_{i}^{baseline}$ denote the frequencies of TCR clonotype *i* in the Biotin^+^ group and the baseline group, respectively. Similarly, $p_{irrelevant}^{Biotin}$ and $p_{irrelevant}^{baseline}$ represent the total frequencies of irrelevant TCRs in the Biotin^+^ and baseline groups, respectively. The *S*_*i*_ represents the potency score for a given clonotype *i*, which can be calculated by:

$$S_{i}=\frac{p_{i}^{Biotin}}{p_{i}^{baseline}}\;\cdot \;\frac{\sum p_{irrelevant}^{baseline}}{\sum p_{irrelevant}^{Biotin}}$$

This formulation ensures that *S*_*i*_ reflects the relative enrichment of a clonotype in the Biotin^+^ group, independent of its baseline abundance. By normalizing against the variability in irrelevant TCR distributions, this calculation ensures that the potency score *S*_*i*_ is robust and comparable across samples and experimental conditions. Additionally, to ensure accurate potency score calculations and minimize noise from ultra-low-frequency clonotypes, we restricted the calculation of potency scores to clonotypes with baseline frequencies above a specified cutoff. The cutoff was determined based on the method used to estimate baseline frequencies: 0.1% for estimates derived from scTCR-seq data and 0.01% for those derived from bulk TCR-seq data. For CMV-specific clonotypes, baseline frequencies were established through bulk TCR-seq.

### Bulk TCR-seq data analysis

TCR-seq data were processed with Cogent NGS Immune Profiler (version 1.0) obtained from Takara Bio under default parameters. TCR clonotypes with any frameshift or stop codon were excluded from downstream analysis. Subsequently, the frequencies of TRA clonotypes and TRB clonotypes were then scaled to sum to 1, respectively. To simplify the process and facilitate integrated analysis with the single-cell data, clonotypes were defined at the amino acid level, with CDR3 sequences sharing identical amino acid composition collapsed into a single clonotype for analysis.

### Analysis of PRECISE-seq data from the tumor model

The scRNA-seq reads were aligned to the mm 10 genome using the “cellranger count” command, while scTCR-seq reads were aligned to the mouse GRCm38 V(D)J reference (version 7.0.0) using the “cellranger vdj” command. Then, the PRECISE-seq data were processed as previously described. Cells of low quality, characterized by the expression of <300 genes or > 15% mitochondrial gene expression, were removed. Clusters exhibiting a low rate of TCR detection or the expression of non-T cell markers were also removed. HVGs were defined as the top 2,500 variable genes, excluding the TCR variable region genes. Next, cell-cycle scores were assigned based on the expression of G2/M- and S-phase markers, using the CellCycleScoring function in the Seurat package. An interferon-response score was also assigned to each cell based on the expression of the HALLMARK_INTERFERON_ALPHA_RESPONSE gene signature from the MSigDB (Liberzon et al., 2015). The unwanted effects caused by the cell cycle, total UMI counts, the percentage of mitochondrial genes, and the interferon-response signature were removed by regression. The Biotin^+^ samples and the baseline samples were integrated using the integration procedure in the Seurat R package, with baseline samples set as the reference. Subsequently, the Seurat pipeline was utilized to identify cell clusters using the Louvain algorithm. Clusters exhibiting high levels of cell-cycle scores or interferon-response scores were removed. Then, we applied the Seurat clustering pipeline again to the filtered high-quality cells.

### TCR repertoire analysis of PRECISE-seq data from tumor models

The TCR data from PRECISE-seq were analyzed following the methods previously described, with certain modifications. Briefly, cells with only a single TCR chain detected were also retained in order to increase the number of available cells for the analysis. Clonotypes with more than two TCRα or TCRβ chains were considered artifacts resulting from doublets and were excluded from the downstream analysis. Considering that tumor-specific TCRs generally exhibit lower affinity than viral-specific TCRs (Aleksic et al., 2012), the thresholds for defining high-confidence antigen-specific clonotypes were adjusted. Specifically, high-confidence tumor-specific clonotypes in the Biotin^+^ group were defined as those meeting the following criteria: (1) a minimum cell number threshold of 2; (2) a P value <0.1; and (3) an OR >2 compared with the baseline group. The potency score for each clonotype was calculated as previously described, using the frequencies determined by scTCR-seq data.

### Trajectory interference of tumor-infiltrating T cells

Two distinct algorithms, URD and diffusion map, were utilized to infer the developmental trajectories of CD8^+^ T cells from the PRECISE-seq data generated using the MC38 model. To infer the differentiation origin of the T_Ly49_ subset, a tree-structured trajectory was constructed using the URD R package (version 1.1.1) (Farrell et al., 2018). Three precursor subsets (T_N_/T_CM_, T_PEX_, and T_EM_) were selected, with T_N_/T_CM_ designated as the root of the tree, while T_EX_ and T_Ly49_ subsets were assigned as the tips. The top 30 PCs previously calculated using the Seurat package were adopted. Logistic parameters were determined using the pseudotimeDetermineLogistic function with the settings optimal.cells.forward = 20 and max.cells.back = 40. To simulate random walks from all tips, the *n*.per.tip parameter was set to 25,000. The divergence method for constructing the tree structure was specified as “preference.”

In the diffusion map analysis, T_PEX_, T_EM_, T_EX_, and T_Ly49_ subsets from untreated tumors were utilized (Angerer et al., 2016). The differentially expressed genes (DEGs) among these four subsets were identified using the FindAllMarkers function with min.pct = 0.2. These DEGs were then intersected with the HVGs to generate an informative gene list. The expression of these informative genes was extracted from the integrated assay to create a diffusion map with the settings n_pcs = 20, n_eigs = 20, and sigma = 3.

### Analysis of scRNA-seq data from cancer patients undergoing immunotherapy

The public scRNA-seq datasets were analyzed with the Seurat package. For scRNA-seq dataset of MSI CRC cohort from Li et al. (2023), 27 tumor samples from 19 patients were analyzed. These patients were treated with toripalimab (αPD-1 Ab) with or without celecoxib before curative surgical resection. Responders were defined as patients who achieved a pathological complete response, while nonresponders were defined as patients who did not achieve a pathological complete response. The processed data were downloaded from the GEO database. The cell barcodes for CD8^+^ T cells were obtained from Table S2 of the original publication. Gene expression normalization was carried out as previously described. HVGs were selected for individual patients with FindVariableFeatures function in the Seurat package, and were further combined using the SelectIntegrationFeatures function. The HVGs were then scaled using the ScaleData function.

For the scRNA-seq dataset of the CRC cohort from Chen et al. (2024), 44 tumor samples from 22 patients were analyzed. The processed data were downloaded from the GEO database with the accession number GSE236581. These patients received systematic anti-PD-1 immunotherapy and the following surgery if necessary. Responders were defined as patients who achieved a complete response. For the scRNA-seq dataset of HBV^+^ HCC cohort from Guo et al. (2025), 14 tumor samples from 9 patients were analyzed. The processed data were downloaded from the GEO database with the accession number GSE235863. These patients received the combination therapy of anti-PD-1 and lenvatinib without prior systemic treatment. Responders were defined in the original publication as including both complete and partial responses. For the scRNA-seq dataset of the melanoma cohort from Sade-Feldman et al. (2018), 45 tumor samples from 32 patients were analyzed (one sample was excluded due to a low number of CD8^+^ T cells available for the analysis). We analyzed the patients who were treated with αPD-1 Ab with or without αCTLA-4 Ab. Two samples from patients who only received αCTLA-4 Ab treatment were excluded. The normalized gene expression matrix was downloaded from the GEO database. The cell names for CD8^+^ T cells were obtained from Table S2 of the original publication. We selected and scaled the HVGs and then performed the clustering procedure to exclude a small subset of B cells.

To assign a cellular state for each CD8^+^ T cell in the public datasets, we first construct a reference profile for each CD8^+^ T cell subset identified in the PRECISE-seq data of the mouse tumor model. To enhance discrimination, the T_PEX_ subset was merged into the T_EX_ subset. Then, the marker genes for each subset were identified from PRECISE-seq data using the FindAllMarkers function. The informative genes were defined as the intersection of marker genes and HVGs. Subsequently, the scaled data of informative genes, stored in the “integrated” assay, were utilized to calculate the average profile of each CD8^+^ T cell subset. The mouse gene symbols of informative genes were then converted to their human one-to-one orthologs. The converted informative genes were further filtered based on their intersection with the HVGs of each query dataset. The Pearson correlations between each CD8^+^ T cell from public datasets and the reference profiles were calculated using the scaled data of these genes. The cellular state for each CD8^+^ T cell was assigned based on the most similar reference profile. The T_EM_/T_EFF_-to-T_Ly49/KIR_ cell ratio based on the assigned cellular state was used to split samples into two groups (ratio > 1 and <1). The Kaplan–Meier survival analysis was subsequently employed to assess the association between these groups and survival rates, with the survminer R package utilized for visualization.

### Statistical analysis

Statistical analysis was performed using GraphPad Prism 8 (GraphPad Software). Unless otherwise noted in the figure legends, each dot represents a biological replicate. Data are presented as the mean ± SEM. Comparisons of two groups were performed by using two-tailed paired Student’s *t* test unless otherwise indicated, ns, P > 0.05; *P < 0.05; **P < 0.01; ***P < 0.001; or ****P < 0.0001. Fisher’s exact test was performed with Fisher’s test using MATLAB R202la. Information on specific statistical tests is listed in the figure legends and/or method details.

### Online supplemental material

Fig. S1 shows the development of PRECISE‑seq and its capacity to label cell–cell contacts and antigen‑specific interactions. Fig. S2 shows the characterization of CMV‑specific human CD8⁺ TCR repertoires and phenotypes captured by PRECISE‑seq. Fig. S3 shows in vivo monitoring of tumor‑specific T cells and the identification/validation of tumor‑reactive TCRs. Fig. S4 shows phenotypic and clonotypic features of T_Ly49_ cells compared with other CD8⁺ T cell states. Fig. S5 shows how tumor‑specific T cells skew toward a regulatory T_Ly49_ program and how αPD‑1 therapy reshapes these states. Tables S1 and S2 list peptide sequences and hashtag Abs used for single‑cell sequencing.

## Supplementary Material

Table S1 shows the list of peptide sequences.

Table S2 shows the list of hashtag Abs used for single-cell sequencing.

## Data Availability

The PRECISE-seq datasets reported in this paper have been deposited in the Genome Sequence Archive (GSA) (Chen et al., 2021) in the National Genomics Data Center (CNCB-NGDC Members and Partners, 2022), China National Center for Bioinformation/Beijing Institute of Genomics, Chinese Academy of Sciences. The human dataset has been deposited in the GSA-Human database with an accession number HRA006819 that is publicly accessible at https://ngdc.cncb.ac.cn/gsa-human. The mouse dataset is deposited in the GSA database with an accession number CRA015233 and can be found at https://ngdc.cncb.ac.cn/gsa. All other materials will be made available upon request.
