# Supplementary material for: PRECISE-seq reveals disease-relevant TCR repertoires with phenotypic plasticity
Source: J Exp Med. 2026 May 12;223(6):e20251779. doi: 10.1084/jem.20251779 (PMC13165028; doi:10.1084/jem.20251779)
Supplement: Table S1 — shows the list of peptide sequences. [file jem_20251779_tables1.docx]

Table S1. List of peptide sequences

| Peptide name | Peptide sequence | Note |
| --- | --- | --- |
| Biotin-LPETG*G probe | Biotin-AALPET-(2-Hydroxyacetic acid)-G | G* refers to 2- hydroxyacetic acid |
| N4 (OVA_257-264_) | SIINFEKL |  |
| Q4 | SIIQFEKL |  |
| T4 | SIITFEKL |  |
| Q4H7 | SIIQFEHL |  |
| G4 | SIIGFEKL |  |
| E1 | EIINFEKL |  |
| LCMV gp_33-41_ | KAVYNFATM |  |
| CMV pp65_495-503_ | NLVPMVATV |  |
| NY-ESO-1_157-165_ | SLLMWITQC |  |
| AP-HA probe | GGGGGYPYDVPDYASSC |  |
