# Supplementary material for: PRECISE-seq reveals disease-relevant TCR repertoires with phenotypic plasticity
Source: J Exp Med. 2026 May 12;223(6):e20251779. doi: 10.1084/jem.20251779 (PMC13165028; doi:10.1084/jem.20251779)
Supplement: Table S2 — shows the list of hashtag Abs used for single-cell sequencing. [file jem_20251779_tables2.docx]

Table S2. List of Hashtag Ab used for single-cell sequencing

| **Markers** | **Barcode** | **Cat** | **RRID** |
| --- | --- | --- | --- |
| TotalSeq-C0312 anti-mouse Hashtag 12 Antibody | CTGCAAATATAACGG | 155883 | AB_2924483 |
| TotalSeq-C0313 anti-mouse Hashtag 13 Antibody | CTACATTGCGATTTG | 155885 | AB_2922483 |
| TotalSeq-C0314 anti-mouse Hashtag 14 Antibody | CTTTCGCCAACTCTG | 155887 | AB_2922484 |
| TotalSeq-C0315 anti-mouse Hashtag 15 Antibody | CCCTCTCTGGATTCT | 155889 | AB_2924484 |
| TotalSeq-C0254 anti-human Hashtag 4 Antibody | AGTAAGTTCAGCGTA | 394667 | AB_2801034 |
| TotalSeq-C0263 anti-human Hashtag 13 Antibody | AAATCTCTCAGGCTC | 394685 | AB_2904414 |
